# Advancements in Battery Materials: Bio-Based and Mineral Fillers for Next-Generation Solid Polymer Electrolytes

**DOI:** 10.1021/acsami.4c11214

**Published:** 2024-11-07

**Authors:** Tayyab Subhani, Sanaz Khademolqorani, Seyedeh Nooshin Banitaba, Mohamed Ramadan, Abdul Khaliq, Imran Ali Chaudhry, Ahmed I. Osman

**Affiliations:** † College of Engineering, University of Ha’il, P.O. Box 2440, Hail 81481, Saudi Arabia; ‡ Emerald Experts laboratory, Isfahan Science and Technology Town, Isfahan 84156-83111, Iran; § Central Metallurgical Research and Development Institute (CMRDI), P.O. Box 87, Helwan 11421, Egypt; ∥ School of Chemistry and Chemical Engineering, Queen’s University Belfast, Belfast BT9 5AG, Northern Ireland, U.K.

**Keywords:** Li-ion batteries, Solid-state polymer electrolyte, Natural filler, Biobased filler, Interfacial engineering, Mineral filler

## Abstract

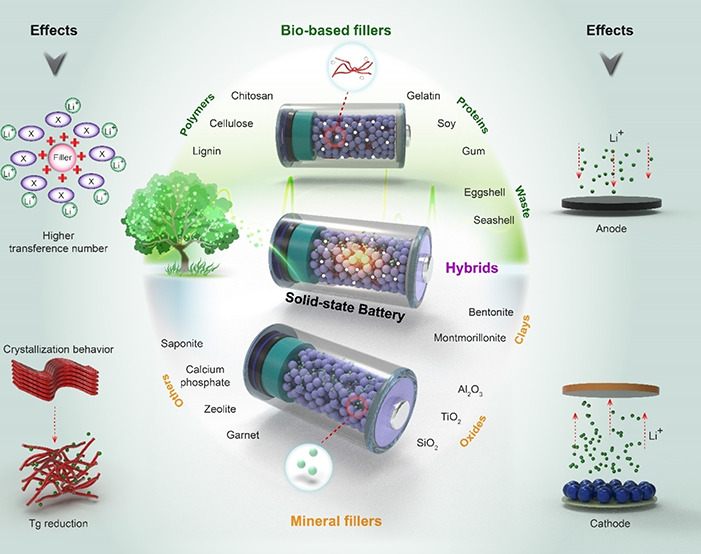

The state-of-the-art all-solid-state batteries are expected to surpass conventional flammable Li-ion batteries, offering high energy density and safety in an ultrathin and lightweight solvent-free polymeric electrolyte (SPE). Nevertheless, there is an urgent need to boost the room-temperature ionic conductivity and interfacial charge transport of the SPEs to approach practical all-solid-state devices. Accordingly, loading filler grains into SPEs has been well-documented as a versatile strategy, promoting the overall electrochemical performance. In this era, using natural resources to extract filler additives has attracted tremendous attention to curb fossil fuel dependency. Also, there is a growing preference for materials that impose minimal environmental harm, are sustainable, and exhibit environmentally friendly characteristics. Therefore, mineral and biobased fillers, as natural-based additives, are strong candidates to replace traditional petroleum-based synthetic materials. Herein, we conduct a systematic investigation into the ion-transport mechanisms and fundamental properties of the filler-loaded SPEs. Additionally, recent advances in SPE architectures through embedding mineral and biobased fillers, as well as their hybrid compositions, are focused. Finally, the downsides and future directions are highlighted to facilitate further development and research toward revitalizing rechargeable battery-related technology. Overall, efficient methods for modifying SPEs through the use of natural resource organic and inorganic fillers are discussed, and technological advancements and related challenges are emphasized. Following the provided rational solutions to overcome major obstacles faced by SPEs, we hope to meet the demands of a greener future.

## 1 Introduction

Before the onset of the initial Industrial Revolution in the early 19th century, traditional biomass such as wood and coal was the primary source of global energy consumption.^1^ The 1930s marked significant progress in the petroleum industry, setting the stage for a shift from biomass to high-energy petroleum-based resources. The uneven distribution of petroleum reserves led to a surge in demand for petroleum-based energy from regions with limited reservoirs, ultimately culminating in the oil crisis of the 1970s.^2^ This geopolitical situation prompted nations in the global north to seek alternative technologies to revolutionize the energy landscape and reduce the dependence on fossil fuels. Prior to the early 1970s, numerous efforts were made to deploy lithium metal-based high-energy batteries.^3^ Meanwhile, challenges related to the electrically unstable lithium metal electrodeposition formation resulted in the creation of dendrites during cycling, which caused significant safety concerns.

The progression in Li-based battery technology entails a shift from the liquid state to the solid-state, focusing on achieving enhanced energy density, safety features, and intelligent functionalities. Accordingly, solid polymer electrolytes (SPEs) have emerged as an up-and-coming alternative for ion-conducting materials in energy storage and conversion mechanisms.^4^ SPEs offer heightened stability and significantly improved safety compared to organic liquid electrolytes. These configurations are progressively gaining recognition within the scientific community as an evolving category of solid-state ionic conductors. Despite several declared pros for SPE networks, liquid electrolytes often demonstrate superior ionic conductivities compared to polymer electrolytes, motivating researchers to design integrated SPEs with advanced electrochemical features. In this era, ongoing efforts are indispensable in improving SPEs across various dimensions, optimization of interfaces between SPEs and electrodes, lithium-ion (Li-ion) transference enhancement, refinement of electrochemical and thermal stabilities, and particularly encompassing ionic conductivity augmentation to extend longevity.^5^

So far, various strategies have been reported to enhance the ion conductivity of SPE structures, including utilizing high-conductivity polymers, adding plasticizers, and incorporating filler grains. As we explore new materials for enhancing the ionic conductivity and stability of SPEs, the principles of low-energy catalytic processes outlined recently could be pivotal. The integration of such catalytic strategies into SPE manufacturing processes may lead to significant improvements in performance and sustainability. The selection of the polymer in the electrolyte exerts a significant influence on its ion conductivity. Polymers with high dielectric constants and low glass transition temperatures typically manifest elevated ion conductivities. Therefore, poly(ethylene oxide) (PEO), poly(vinylidene fluoride) (PVDF), and polyacrylonitrile (PAN) have been well-documented as the most appropriate polymer matrices. The inclusion of small-molecule plasticizers into polymers augments the flexibility and diminishes the glass transition temperature of SPEs, thus, optimizing ion conductivity. Moreover, loading organic or inorganic fillers to polymeric electrolytes can amplify their ion conductivity, regarding the capability of fillers to impart more disoriented polymer chains in the SPEs, thereby facilitating the migration of Li^+^ ions between the electrodes.^3^Figures S1 and S2 represent the bibliographic coupling map of Scopus-indexed papers related to the keywords solid-state Electrolyte and lithium-ion battery, as well as the number of publications regarding the use of filler-loaded SPEs in battery structures. The provided illustrations display the growing interest of researchers in promoting the features of SPEs through incorporating fillers.

Although both organic and inorganic filler additives can be easily synthesized, there is a growing emphasis on developing sustainable materials in light of technological advancements and heightened environmental consciousness. In several reports, using mineral and biobased additives has been acknowledged as an efficient green methodology to enhance the electrochemical performance of SPEs. Although many studies have overviewed the impact, role, and mechanism of fillers on improving the electrochemical performance of LiBs, there is no comprehensive reference highlighting the effect of environmentally friendly natural-based fillers on the SPE architectures.^6^ This paper aims to pioneer an inquiry into the effects of mineral and biobased additives and their synergistic impacts on enhancing the performance of SPEs. The main objective is to facilitate the use of natural resource fillers to modify the SPE structure in a greener route. The elucidation of the benefits and drawbacks associated with each additive, coupled with an in-depth understanding of their mechanisms and impact on the enhancement of solid polymer electrolytes, is expected to catalyze new avenues of inquiry within the research community operating in this domain.

## 2 The Role of Filler in Enhancing the Electrolyte Performance

**Figure 1:**
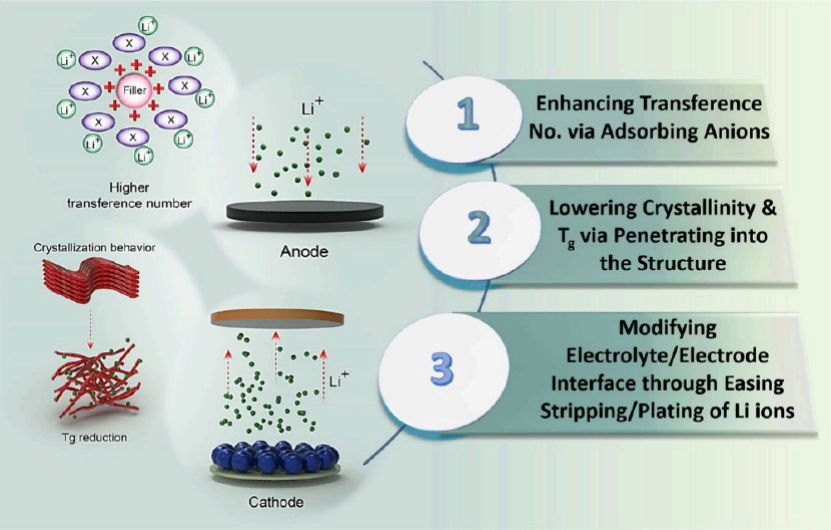
Schematic illustration of the effect of filler on the characteristics of SPEs.

So far, numerous attempts have been made to screen ion transport in solid-state electrolytes. Commonly, this critical issue is explored through electrochemical analysis. Meanwhile, the joint usage of geometrical analysis and bond valence method,^7^ bond valence-Ewald method,^8^ combining the effective medium theory (EMT) and random resistance model (RRM),^9^ and machine learning^10^ have also been declared as other informative methods to discover ion transport in electrolyte networks. SPE structures mainly comprise a lithium salt dispersed in a polymeric host matrix. Commonly, semicrystalline polymers, which comprise crystalline and amorphous regions, are employed as the SPE matrix. The polymer chains of the amorphous sections can freely move above the glass transition temperature (*T*_g_), leading to the motion of free Li^+^ ions in the vacant spaces of the polymer host. In contrast, the crystalline regions endow mechanical integrity and limit the Li^+^ ion movements below *T*_g_. Accordingly, the poor ionic conductivity of the SPEs could be effectively addressed by diminishing the crystallinity and expanding the amorphous domains. Based on this prevailing strategy, filler particles have received significant attention to enhance the ionic conductivity of SPEs. Filler elements mainly affect the formation of amorphous regions and the dissociation of lithium salt ion pairs. When the fillers are introduced into the SPE networks, the filler component penetrates between the polymer chains and increases the number of amorphous phases in the polymeric structure. In addition, the dissociation of lithium salt segments could be boosted via the presence of fillers, causing the generation of more free anions via Lewis acid–base interactions. This results in more free Li^+^ ions forming, enhancing ionic conductivity. Besides, filler particles can significantly improve the mechanical strength of SPEs by serving as a physical reinforcement and creating a more robust and resilient structure. These additives also help disperse stress and reduce crack propagation, ultimately promoting the mechanical durability in SPEs. Furthermore, certain fillers, such as ceramic nanoparticles, can expand the electrochemical stability window by mitigating side reactions and facilitating the transfer of Li^+^ ions. In essence, these additives mitigate potential side reactions at the electrode–electrolyte interface, thereby extending the operational voltage range. Moreover, some fillers can improve Li^+^ ion transfer kinetics due to their inherent ion conductivity. Figure 1 schematically displays the main roles of filler grains in the enhancement of the electrochemical performance in SPEs.

**Figure 2:**
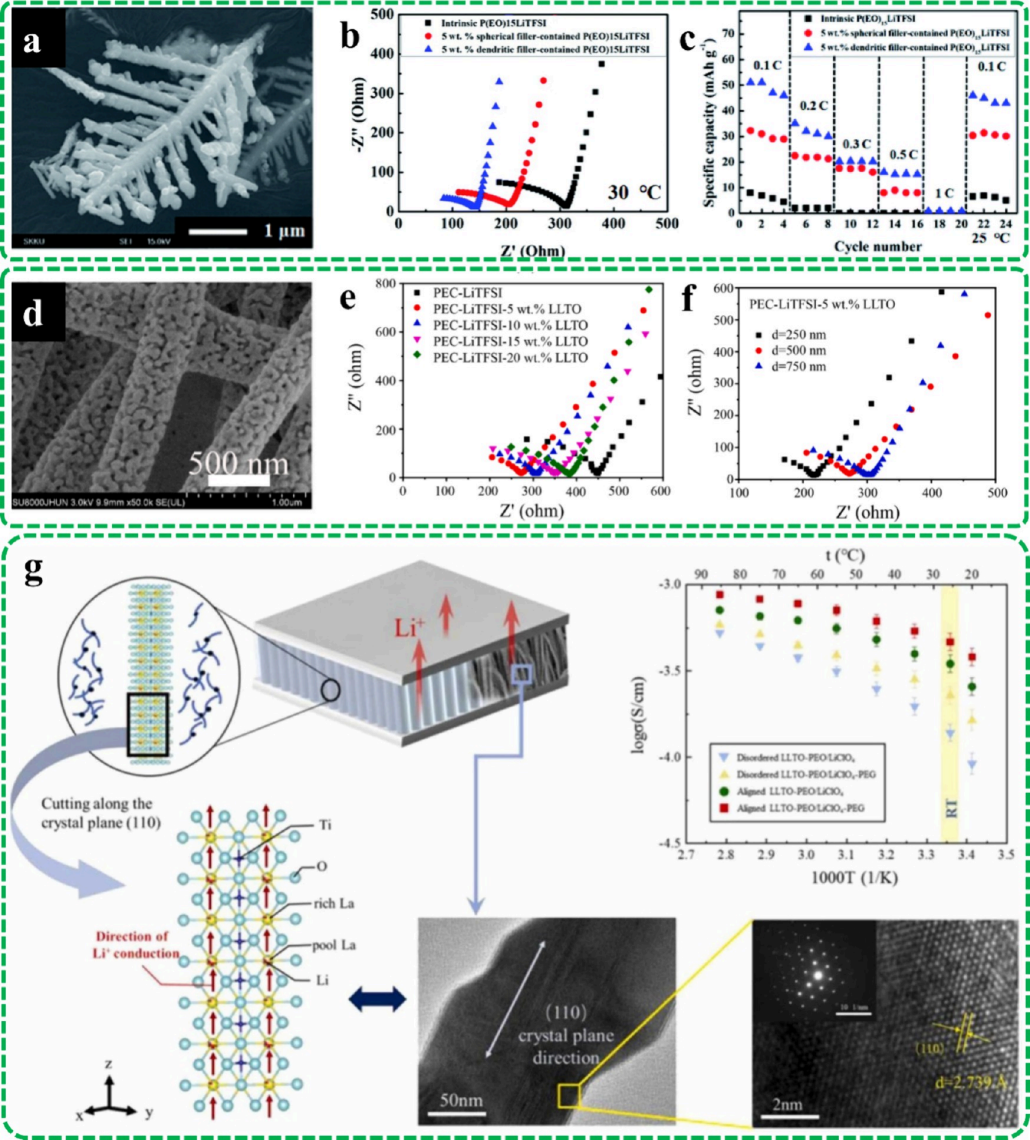
Role of filler morphology in enhancing the ion conductivity in SPEs: (a) SEM image of synthesized dendritic copper oxide powder, (b) Ion conductivity, and (c) Specific discharge capacity of the filler-free SPE in a Li|LiFePO_4_ cell as well as loaded sample with spherical and dendritic powders. Reproduced from ref (12). Available under a CC-BY NC 3.0 license. Copyright [2019] Choi et al. PVC-based SPE loaded with LLTO electrospun fibers: (d) SEM image of the LLTO nanofibers, (e) ion conductivity of the designed SPE embedded with various filler ratios, and (f) the impact of filler diameter on the ion conductivity. Reproduced with permission from ref (13). Copyright [2021/Elsevier]. Loading oriented LLTO nanofibrous filler into an SPE structure: (g) Schematic illustration of the ion conductivity in the designed SPE. Reproduced with permission from ref (14). Copyright [2022/Elsevier].

Filler components could be more influential depending on their morphology, formation, distribution, size, and type. Changes in the morphology of filler additives may affect their specific surface area, promoting the electrochemical performance of the generated composite.^11^ For example, Choi et al.^12^ compared the effect of spherical and dendritic copper oxide powder on various characteristics of the generated PEO-based SPE. The spontaneous galvanic displacement reaction was used to synthesize dendritic formations of the filler (See Figure 2a). The addition of spherical and dendritic particles led to a reduction in the recrystallization from 24 to 13.89 and 13.43%, respectively. The shift of the powder morphology from spherical to dendritic causes more interaction with the polymer matrix. Ion conductivity was also inclined from 0.3623 × 10^–1^ mS·cm^–1^ to 0.7258 × 10^–1^ and 1.007 × 10^–1^ mS·cm^–1^ by embedding 5 wt % spherical and dendritic powders (See Figure 2b). The observed difference between the obtained ion conductivities in the filler-loaded samples could be assigned to superior surface area and more interaction with the polymer matrix. As a result of higher ionic conductivity, the electrochemical cell comprising the dendritic filler powder exhibited reasonable and superior specific capacities in various C-rates even at room temperature (See Figure 2c). In another work, Zhao et al.^13^ added the Li_0.33_La_0.557_TiO_3_ (LLTO) electrospun fibers as filler into a PVC-based SPE. An SEM image of the fabricated filler is illustrated in Figure 2d. LLTO nanofibers could provide higher ionic conductivity compared with the LLTO particles due to acting as Li^+^ conductive tunnels between the electrodes. The results showed that loading LLTO up to 5 wt % could lead to enhancing the ionic conductivity from 3.86 × 10^–2^ to 6.21 × 10^–2^ mS·cm^–1^ while exceeding this amount caused a reduction in the ion conductivity might be due to the filler aggregation (See Figure 2e). A reduction in the fiber diameter also affected the electrochemical performance by increasing the ion conductivity as well as the electrochemical stability window (See Figure 2f). This could be linked to the placing of a higher number of LLTO fibers between the polymer chains and so favoring the transient cross-linking breaking of the polymer chains.

Additionally, finer fibers possess superior specific areas, creating more Li^+^ ions. Moreover, fine fibrous fillers occupy a more working electrode surface, preventing the oxidation of polymer matrix against lithium metal. Following this paper, Li et al.^14^ illustrated that the vertical orientation of the LLTO nanofibers in the SPE structures could further boost the ionic conductivity up to 4.67 × 10^–1^ mS.cm^–1^. The presence of aligned conductive channels in the SPE structure accelerates the transfer of Li^+^ ions between the electrodes, boosting the ion conductivity (See Figure 2g).

Yap et al.^15^ also analyzed the effect of filler size on the final electrochemical performance. It was reported that the addition of alumina in the micron range (<10 μm) and nano size (<50 nm) could lead to an enhancement in the ionic conductivity from 1.701 × 10^–5^ to 2.970 × 10^–5^ and 4.843 × 10^–6^, respectively. Based on the obtained outcomes in this attempt, the filler powder with a larger diameter could be more effective than the finer particles, possibly due to more abundant filler grains, which can immobilize the long polymer chains. This led to a decrease in the conduction pathways and reduced ionic conductivity. Despite the shape, size, and dispersion of the fillers, the filler type and its concentration significantly affect the electrochemical properties of the resulting SPE network.

Filler particles offer significant benefits for enhancing the performance of SPEs. However, achieving a consistent and stable dispersion of these particles remains a major challenge, preventing their full potential from being realized. This challenge can be attributed to filler aggregation and poor compatibility between the filler and the polymer. Filler particles often aggregate due to strong van der Waals forces, and the mismatch between the surface energies of the particles and the polymer matrix can also contribute to aggregation and phase separation. Therefore, proper dispersion of fillers within the SPE structures is critical for enhancing their mechanical, thermal, and electrochemical properties. An even distribution of fillers ensures optimal ionic conductivity, structural integrity, and overall performance. This could be controlled through employing various strategies, including solution mixing, melt mixing, electrospinning, the use of chemical modification, ultrasonication, the control of filler loading, and drying and curing techniques. In virtue of the solution mixing technique, fillers could be dispersed in a solvent via vigorous stirring and sonication. In addition, surfactants or dispersants could be employed to enhance the wettability of the fillers and prevent particle agglomeration. Regarding the melt mixing method, the polymer and fillers are mixed in the molten state using extruders, resulting in a proper dispersion through shear forces that break up the agglomerates. Electrospinning could also be employed to create nanofibers containing uniformly dispersed fillers. The high electric field helps stretch and align the polymer solution, promoting an even distribution of fillers. Chemical treatments of the fillers could also increase their compatibility with polymer matrices, assisting in a better interaction within the polymer. Applying ultrasonic waves can also help break up the agglomerate via generated cavitation in the ultrasonication procedure. Besides, the filler concentration should be optimized. A high filler loading can lead to accumulation, while being too low may not provide sufficient advantages. Furthermore, manipulating the time and temperature during the curing procedure could generate particles with better interaction with the polymer matrices. Considering the growing progress in modeling artificial intelligence progress, utilizing computational simulations could be useful to precisely predict filler dispersion behavior and optimize processing parameters toward approaching better performance.^16^

**Figure 3:**
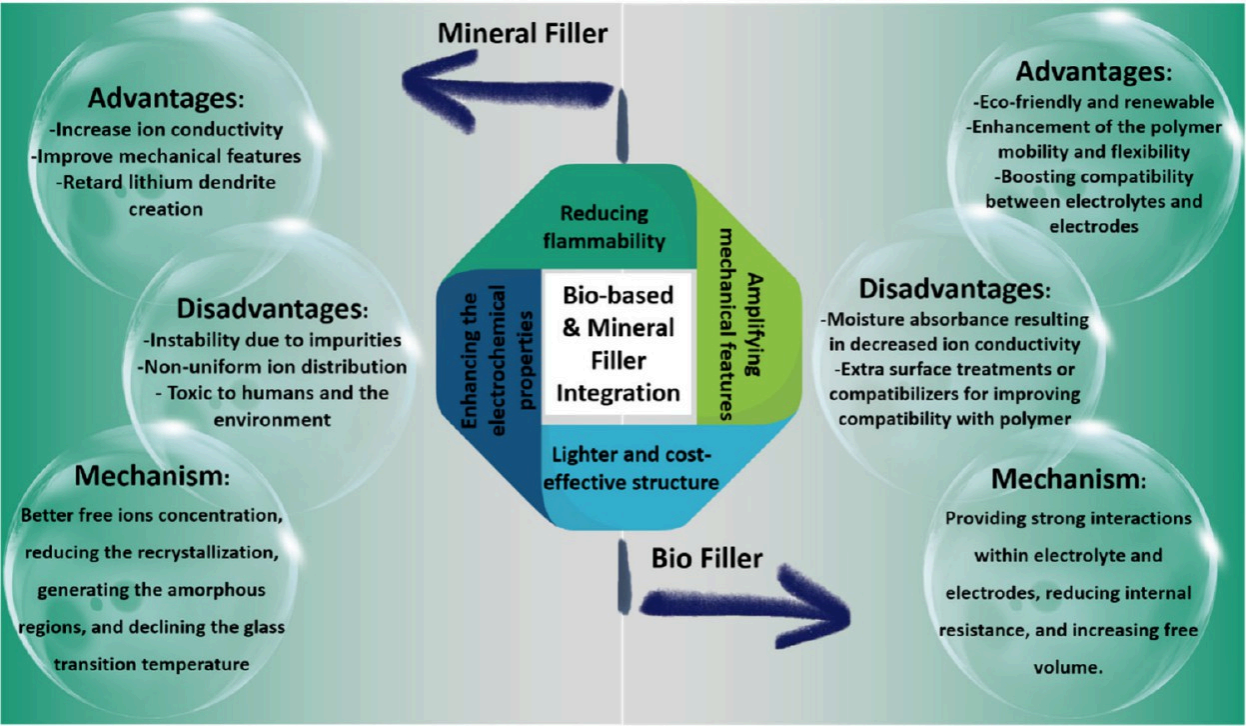
Advantages, disadvantages, and mechanism of mineral and biobased fillers, as well as their compositions for integrating the electrochemical performance of SPEs.

Filler grains display various outcomes, depending on the intrinsic features and interactions with the composite components. The filler additives used for the SPE structures could be categorized based on their resources into two main classes of organic and inorganic materials. Organic fillers are composed of carbon and hydrogen and could be naturally formed or synthetic. Meanwhile, inorganic substances comprise oxide, hydroxide, salt, metal, and silicate subgroups prepared through mining or synthesis procedures. Within the last couple of years, exploring mineral and biobased additives has reaped significant attention to reducing reliance on fossil fuels and synthetic chemicals. The production and disposal procedures of these natural substances generally have a lower carbon footprint. These materials are renewable, biodegradable, and generally pose lower risks of allergic reactions or toxicity. Accordingly, the biobased and mineral fillers as organic and inorganic substances, alongside their combinations, are preferable for developing highly efficient green SPEs. Figure 3 schematically shows the advantages and main mechanisms of the mineral and biobased materials, alongside their compositions as fillers toward enriching the overall performance of the SPEs. Considering the importance of this issue, the usage of mineral-based galleries, biobased grains, and their hybrid compositions as fillers for enhancing the electrochemical performance of the SPEs is comprehensively overviewed in the following sections.

## 3 Mineral-Based Filler-Integrated Polymer Electrolytes

Mineral particles have been regarded as the most promising eco-friendly fillers for enhancing all-solid-state polymeric electrolytes’ electrochemical performance. These elements could be classified into various categories based on their geometry, chemistry, or application. According to the morphological features, the mineral fillers are shaped like spheres, cubes, blocks, flakes, and fibrous architectures. Additionally, based on their chemical compositions, such components could be categorized into four types: oxide, salt, elemental substance, and organic matter. In the SPE structures, oxide blocks and clay flakes are the most favorable fillers, boosting electrochemical efficiency.^17,18^ This section provides recent progress in the SPE domain, focusing on the incorporation of mineral oxides, clay nanoflakes, and other well-known mineral substances.

### 3.1 SPEs Loaded with Mineral Oxides

Mineral oxide fillers, known as inert fillers, are a common category of additives loaded into polymer electrolytes to enhance ionic conductivity.^19^ Al_2_O_3_, TiO_2_, ZrO_2_, and SiO_2_ are regular inert particulate fillers employed to enhance the ionic conductivity at ambient temperature and improve the stability of the electrode–electrolyte interface.^20^ The particulate additives penetrate the polymer chains, resulting in a reduction in the recrystallization, generating the amorphous regions, and lowering the glass transition temperature.^21^ Additionally, the ionic conductivity improves through Lewis acid–base interactions between the O^2–^ or OH^–^ groups of the fillers with the mobile ions, thereby increasing the salt dissociation and Li^+^ ion mobility. Moreover, these solid plasticizers create Li^+^ ion conducting pathways on the surface of particulate fillers, easing the ion transfer between the electrodes. Table 1 summarizes recent approaches to enhancing the ionic conductivity of SPEs by embedding various inert mineral oxide fillers.

**Table 1:** Recent advancements toward obtaining higher ionic conductivity in all-solid-state electrolytes are by loading inert mineral oxide fillers

**Employed polymer matrix**	**Additive type**	**The impact of incorporated additives on SPE’s characteristics**	**Main results and outcomes**	**ref**
PEO	Al_2_O_3_	Excellent ion transport properties and Li-electrolyte interface stability	At an Al_2_O_3_ filler content of 15 wt %, the provided SPE exhibited a room-temperature ionic conductivity of 3.38 × 10^–1^ mS·cm^–1^, an ion migration number of 0.70, and an electrochemical window of 5.6 V.	Li et al.^22^
PEO–PVDF		Supreme ion transport number and upright electrochemical properties	Loading 2 wt % ceramic additive caused an enhancement in the ionic conductivity of membranes up to 1.25 × 10^–1^ mS.cm^–1^, and a rise in the electrochemical stability window up to 4.75 V, due to hindering the formation of PEO crystalline zones.	Wilson et al.^23^
Poly(ether block amide)		Enhancing the cycling performance and ion conductivity	Polymer membrane with 3 wt % Al_2_O_3_ nanoparticles displayed an ion conductivity of 3.57 × 10^–2^ mS.cm^–1^ at 25 °C. Also, the Li symmetrical battery assembled showed excellent cycling stability for 1000 h at 0.1 mA cm^–2^. An all-solid-state structure maintained 94.9% of its maximum capacity even after 650 cycles at 60 °C with an average Coulombic efficiency of 99.84%.	Liu et al.^24^
PEO		Boosting ionic conductivity and cycle performance	The ionic conductivity of the designed electrolyte of up to 2.02 × 10^–1^ mS.cm^–1^ was approached. The electrolyte exhibited a low overpotential change of 0.06 V, Coulombic efficiency above 99.6%, and capacity retention of 95% in the electrochemical cell of Li|SPE|LiFePO_4_, tested at 0.5C and 60 °C for 50 cycles.	Song et al.^25^
PEO	SiO_2_	Heightening electrochemical performance	The electrolyte containing 6 wt % SiO_2_ nanoparticles exhibited a relatively high ionic conductivity of 2 × 10^–1^ mS.cm^–1^ at 60 °C, as well as an electrochemical window of 5.6 V vs Li/Li^+^. Additionally, it presented a tensile strength of 3.63 MPa and a breaking elongation of 911%. The designed cell showed a stable potential of 27 mV over 480 h at 0.2 mA.cm^–2^ current density.	Liu et al.^26^
PEO		Escalating the ionic conductivity	The electrospun PEO-based nanofibers embedded with 0.07 wt % SiO_2_ nanoparticles caused a synergetic effect, resulting in the enhancement of ionic conductivity to 0.0035 mS.cm^–1^.	Banitaba et al.^27^
PEO		Raising electrochemical performance	Adding hollow SiO_2_ into the polymeric electrolyte improved the Li^+^ transference number to 0.49, ionic conductivity about 1.07 × 10^–1^ mS.cm^–1^ at 30 °C, and widened the electrochemical stability window around 4.73 V. The prepared solid-state structure displayed a reversible discharge capacity of 136.95 mAh.g^–1^ even after 300 cycles with 96.79% capacity retention at 0.5 C in a Li|SPE| LiFePO_4_ structure.	Jiang et al.^28^
PEO		Enhancing the Li-ion migration number	The ion migration number increased to 0.282 at room temperature by adding 10 wt % SiO_2_ nanospheres. The proposed structure exhibited a high ionic conductivity of 1.03 mS.cm^–1^. Also, the designed electrolyte showed excellent tensile strength (1.12 MPa), as well as elongation at the break of 488.1%.	Shi et al.^29^
PEO	TiO_2_	Smoothing interfacial stability and ionic conductivity	TiO_2_ microrods improved Li//Li cells to last over 1000 h at 0.2 mA.cm^–2^. The liB with LiFePO_4_ cathodes displayed superior cyclability (162.4 mAh.g^–1^ at 0.33C after 200 cycles) and rate capability (132 mAh.g^–1^ at 2C).	Luo et al.^30^
PEO		Advancing Li-ion Conduction	The ion conductivity of electrolytes with 13 nm nanosized TiO_2_ particle-sized increased to 4.1 × 10^–2^ mS.cm^–1^ at 30 °C. The symmetric batteries with TiO_2_ additives last up to 590 h at 0.25 mA.cm^–2^. Full Li metal batteries, paired with a LiFePO_4_ cathode, last 550 cycles.	Su et al.^31^
PEO		Enhancing room temperature ionic conductivity	TiO_2_ nanoparticles were embedded into the PEO-based electrospun fibers to incline the Li-ion conductivity.	Banitaba et al.^32^
			The highly porous structure of the electrospun fibers accelerated the conductivity of ions and boosted the conductivity up to 0.085 mS·cm^–1^ via loading 0.175 wt % filler.	
PEO		Refining the ionic conductivity	The incorporation of 5.0 wt % TiO_2_ into a composite electrolyte resulted in a marked increase in ionic conductivity of 3.4 × 10^–2^ mS.cm^–1^ at 60 °C, nearly ten times greater than the original cross-linked electrolyte without a filler. The prepared electrolyte displayed an excellent cycle stability of 156 mAh·g^–1^ with 97% Coulombic efficiency after 20 cycles.	Soontornnon et al.^33^

### 3.2 Embedding Clay Flakes into the SPEs

The term “clays” is a common name used for all sedimentary particles. High surface-to-volume ratio, feasible modification, size and dimension adjustability, abundance in the crust of the Earth, and low cost are favorable features of clay structures, which have attracted the attention of researchers. According to the literature, clay minerals could be categorized as silicate-based structures, while they are able to comprise O, Al, or Mg elements as well. Tetrahedral forms of Si–O bands are the main skeletal structures of the clay mineral. Additionally, the clay flakes are formed by tetrahedral and octahedral sheets containing OH and Al ions at the apex and center, respectively. In tetrahedral sheets, four oxygen atoms coordinate a cation, and an infinite hexagonal pattern is formed by attaching tetrahedral sheets via a three-cornered joint. Meanwhile, sharing edges connect the octahedral sheets to shape a hexagonal or quasi-hexagonal symmetry.

The addition of clay minerals to the SPEs influences mechanical, thermal, and electrochemical performances. Large interfacial areas, in tandem with the proper rigidity of clay components, cause mechanical properties to be integrated. In addition, the clay filler enhances thermal properties by preventing the polymer from melting and by causing a delay in thermal degradation. The presence of clay elements reduces the host polymer crystallization by acting as a solid plasticizer. Moreover, clay flakes could be easily polarized due to their high dielectric constant, boosting ion pair dissociation and increasing the proportion of free Li^+^ ions. This leads to an increase in transport features, endowing proper compatibility with lithium-based electrodes. The negative charge of silicates in the clay sheets also minimizes the anion migration, thereby approaching high cation transference number values based on Lewis acid dipole interactions. Based on the theories, vermiculite and montmorillonite have shown superior cation exchange capacities of about 130–210 and 70–120 centimole charge per kilogram among various 2D clay minerals. Therefore, these structures might generate more significant ion transfer numbers for SPEs. Meanwhile, lower cation exchange capacity has been declared for other common clay structures, including halloysite (5–50), kaolinite (3–15), and mica (up to 5).^34^Table 2 summarizes recent studies conducted on the design and characterization of SPEs embedded with clay structures.

**Table 2:** Summarization of increasing the ionic conductivity of all-solid-state polymeric electrolytes via embedding clay structures

**Polymer matrix**	**Clay type**	**Role**	**Outcomes**	**ref**
PEO	Vermiculite	Enhancing ionic conductivity, ion transference number, and mechanical strength	The addition of 10 wt.% to the PEO structure boosted the ionic conductivity from 7.5 × 10^–4^ to 1.89 × 10^–1^ mS.cm^–1^, the ion transference number from 0.125 to 0.47, and the mechanical strength from 23.5 to 44.9 MPa. Accordingly, the designed SPE exhibited a specific capacity of 167 mAh·g^–1^ with 82% capacity retention after 200 cycles.	Tang et al.^35^
PEO/poly (vinylidene fluoride-co-hexafluoropropylene) (PVDF-HFP)		Improving ionic conductivity and ion transference number	Embedding 5 wt % vermiculite to the SPE structure enhanced the ionic conductivity from 6.2 × 10^–2^ to 3.7 × 10^–1^ mS.cm^–1^ and raised the ion transference number to 0.34. The designed cell delivered 159 mAh.g^–1^ discharge capacity and 95.5% capacity retention after 200 cycles.	Luo et al.^36^
PVDF	Montmorillonite	Low-cost and high-safety membrane	The composite electrolyte displayed excellent flame retardancy, high ionic conductivity of 2.28 × 10^–1^ mS.cm^–1^, a wide electrochemical stability window of 4.8 V, a high Li-ion transference number of 0.57, and an upright mechanical strength of 12.58 MPa. LiFePO_4_-based solid-state battery, utilizing this electrolyte, depicted stable cycling with a discharge capacity of 130 mAh.g^–1^ for over 100 cycles at room temperature. This demonstrated the potential of utilizing low-cost and eco-friendly raw clay minerals in high-energy solid-state battery applications.	Zhou et al.^37^
PEO–PMMA		Boosting structural dynamics and ionic conductivity	The ion-dipolar-nanofiller coordination suppressed PEO’s crystalline phase in the NSPE films. The 5 wt % MMT incorporated NSPE film showed an order of magnitude increase in ionic conductivity at room temperature. The NSPE films exhibited a high electrochemical stability window, good reversibility and cyclability, and a total ion transference number close to unity. They have ambient temperature ionic conductivity values of 10^–2^ mS.cm^–1^, making them suitable for ion conductors/separators in rechargeable solid-state Li-ion batteries and various ion-conducting electrochemical devices.	Dhatarwal et al.^38^
PEO		Increasing the ionic conductivity and mechanical strength	The ionic conductivity of the electrospun PEO-based electrolyte was inclined from 0.0096 to 0.0160 mS.cm by embedding 0.21 wt.% nanoclay. Also, the mechanical strength was increased from 0.27 to 0.40 MPa.	Banitaba et al.^27^
PEO		Enhancing structural and electrical properties	Polymer-salt complexes were intercalated into the nanometric clay channel by increasing the gallery height of the clay. The surface of montmorillonite was shifted to a hydrophobic structure using an organic modification, which helps in the intercalation of hydrophobic polymer into MMT. Adding 5 wt % clay enhanced the conductivity by 1 order of magnitude, while the conductivity decreased for higher clay concentrations. A change in the cation environment was observed due to the interaction of polymer with clay layers.	Pradhan et al.^39^
PVDF-HFP		Cation transport is advancing, and Lithium dendrite creation retarding	Exfoliated clay nanosheets in the U-CPCE enhanced ionic conductivity up to 1 mS.cm^–1^, resulting in comparable initial discharge capacity to liquid electrolyte-based cells. The U-CPCE-based LIBs also exhibited excellent cycling performance (around 96% capacity retention after 200 cycles at 0.5 C) due to inhibition of lithium dendrite formation and enhanced Li^+^ transference number.	Jeon et al.^40^
PVDF–PVA		Improving ionic conductivity and electrochemical properties	The electrolyte achieved a higher ionic conductivity of up to 4.31 × 10^–1^ mS·cm^–1^ at room temperature through loading 4.0 wt % MMT. The Li/PVDF–PVA-MMT CSPE/LiFePO_4_ cells showed a high specific discharge capacity of over 123 mAh·g^–1^ and a Coulombic efficiency of 97.1% after 100 cycles.	Ma et al.^41^
PVC	Bentonite	Boosting ionic conductivity	The ionic conductivity was increased from 2.37 × 10^–1^ to 4.86 mS·cm^–1^ via loading 10% bentonite.	Ghufira et al.^42^
PEO		Enhancing ionic conductivity and mechanical strength	The ionic conductivity and Young’s modulus were enhanced from 1.23 × 10^–6^ to 1.81 × 10^–4^ mS·cm^–1^ and 70 to 325 MPa by loading 3 wt % bentonite.	Moreno et al.^43^
PAN	Muscovite	Enhancing the mechanical and electrochemical features	The PAN electrolyte incorporated with 5 wt.% muscovite represented the best mechanical properties. The electrochemical cell could retain 83% of the cycling stability and yield a discharge capacity of 134 mAh·g^–1^.	Gabryelczyk et al.^44^
PEO	Kaolinite	Improving ion conductivity, and mechanical properties	The ion conductivity increased with the exfoliated kaolinite content and reached 6.1 × 10^–2^ mS.cm^–1^ at 20 wt % filler content. An amorphous region around the exfoliated kaolinite was beneficial for Li^+^ ion conduction. The PEO matrix decomposition temperature improved at weight loss of 10 and 50 wt % when exfoliated kaolinite was introduced. The mechanical properties of the PEO matrix improved at 15 wt % exfoliated kaolinite filler content.	Chi et al.^45^
PEO/PVDF	Halloysite	Boosting ionic conductivity and high ion transference number	The designed electrolyte exhibited high ionic conductivity 2.45 × 10^–1^ mS.cm^–1^ and ion transference number at room temperature (0.67 at 25 °C). It could be used in a LiFePO4/SPE-H5/Li battery and a 4.3 V high voltage NCM/SPE-H5/Li battery. The composite material also revealed natural clay minerals as sustainable, low-cost nanoceramic fillers for high-energy-density energy storage. The cell also maintained a discharge capacity of 142 mAh.g^–1^ at a 0.2 C rate after 200 cycles.	Wang et al.^46^

### 3.3 Incorporating other Mineral Structures into the SPEs

Besides inert oxides and clay galleries, there are myriad mineral structures employed as fillers to enhance the electrochemical performance of SPEs. For example, Wen et al.^47^ exhibited that the combination of PEO and saponite as SPE structure could lead to the increment of the ionic conductivity from 2 × 10^–5^ mS.cm^–1^ (pure PEO) to 2.1 mS.cm^–1^. Based on the results obtained through this study, the saponite intercalation period could be an effective parameter in determining the ionic conductivity. Therefore, a rise in the intercalation period from 1 to 2h could enhance the ionic conductivity from 2.1 to 4.1 mS.cm^–1^, resulting from placing the PEO polymer chains between the saponite layer. Meanwhile, further increment in the intercalation time (5h) led to a reduction in the ionic conductivity to 1.5 mS.cm^–1^, possibly because of locating extra PEO polymer chains and so deficiency of lithium ions. In another work, Stephane et al.^48^ reported the enhancement of ionic conductivity by loading calcium phosphate to a PEO-based film, concomitating the role of PO^–3^_4_ as a cross-linking center for the PEO, which results in the reorganization of the polymer chains. Also, the embedded filler particles incline ionic transportation by providing low-energy pathways on the surface.

Intending to use the advantages of zeolite-based additives, Lei et al.^49^ synthesized the zeolitic imidazolate framework-8 (ZIF-8) nanoparticles with particle size around 80 nm on a PEO-based SPE structure. In accordance with the schematical mechanism of PEO/ZIF-8, the ZIF-8 nanofillers prevent the restructuring of PEO chains and adsorb anions of lithium salt benefiting from a Lewis acid–base surface. This phenomenon leads to a decrease in the crystallinity of the PEO host and amplifies the dissociation of lithium salt ion pairs. Therefore, the heightened presence of free lithium ions and the polymer amorphous region significantly contribute to the augmentation of ionic conductivity. Also, the minimal solvent content within the porous ZIF-8 filler has the potential to further enhance ionic conductivity. Moreover, the robust interaction between ZIF-8, featuring a Lewis acid surface, and TFSI^–^ anions effectively constrains anion mobility, thereby bolstering lithium cation conductivity and mitigating anion concentration polarization. The presence of ZIF-8 directly affected the mechanical properties of the designed electrolyte by two times throughout all three regions, including prenecking, necking, and breaking region. Additionally, ZIF-8 acted as a cross-linking point to disperse and transfer stress to other polymer chains, which could inhibit Li dendrite growth. Also, the introduction of ZIF-8 increased the Li-ion transference number of the PEO-based SPEs from 0.18 to 0.36 at 60 °C. Additionally, the ionic conductivity at 30 °C was enhanced from 3.6 × 10^–3^ to 2.2 × 10^–2^ mS·cm^–1^. According to the cycle stability, the capacity increased in the initial cycles due to the activation procedure that occurred. The cell retained 111 mAh·g^–1^ discharge capacity and 85% of the capacity after 350 cycles, which was superior to that of the control.

The garnet-type electrolyte is widely regarded as a highly promising solid-state electrolyte for batteries, offering potential advantages in electrochemical stability, energy density, thermal stability, and safety. NASICON and LISICON are two main garnet-type structures, offering promoted ion transport and high ionic conductivity. The ion migration in such structures could be modified by using simulation techniques. Based on the literature, the migration channels and bottlenecks in such structures could be estimated by using hierarchical ion transport algorithms. Cavities and possible migration channels could be evaluated by using geometric analysis. Moreover, the bond valence site energy method can describe migration channels and their corresponding energy barriers. The identification of barriers in migration channels enables the observation of pathways energetically. As a result of modeling a static crystal structure, these methods provide less precision. Meanwhile, ab initio molecular dynamics (AIMD) simulates the ion migration dynamically. Accordingly, it allows the representation of the migration channels in the lattice, identifying the site occupancy, and exploring the jump events.^50^ Additionally, simulations have shown approaching the electrochemical stability window of up to 6 V in the garnet-type electrolytes through exploring the electronic conductivities of all direct and indirect decomposition products.^51^

**Figure 4:**
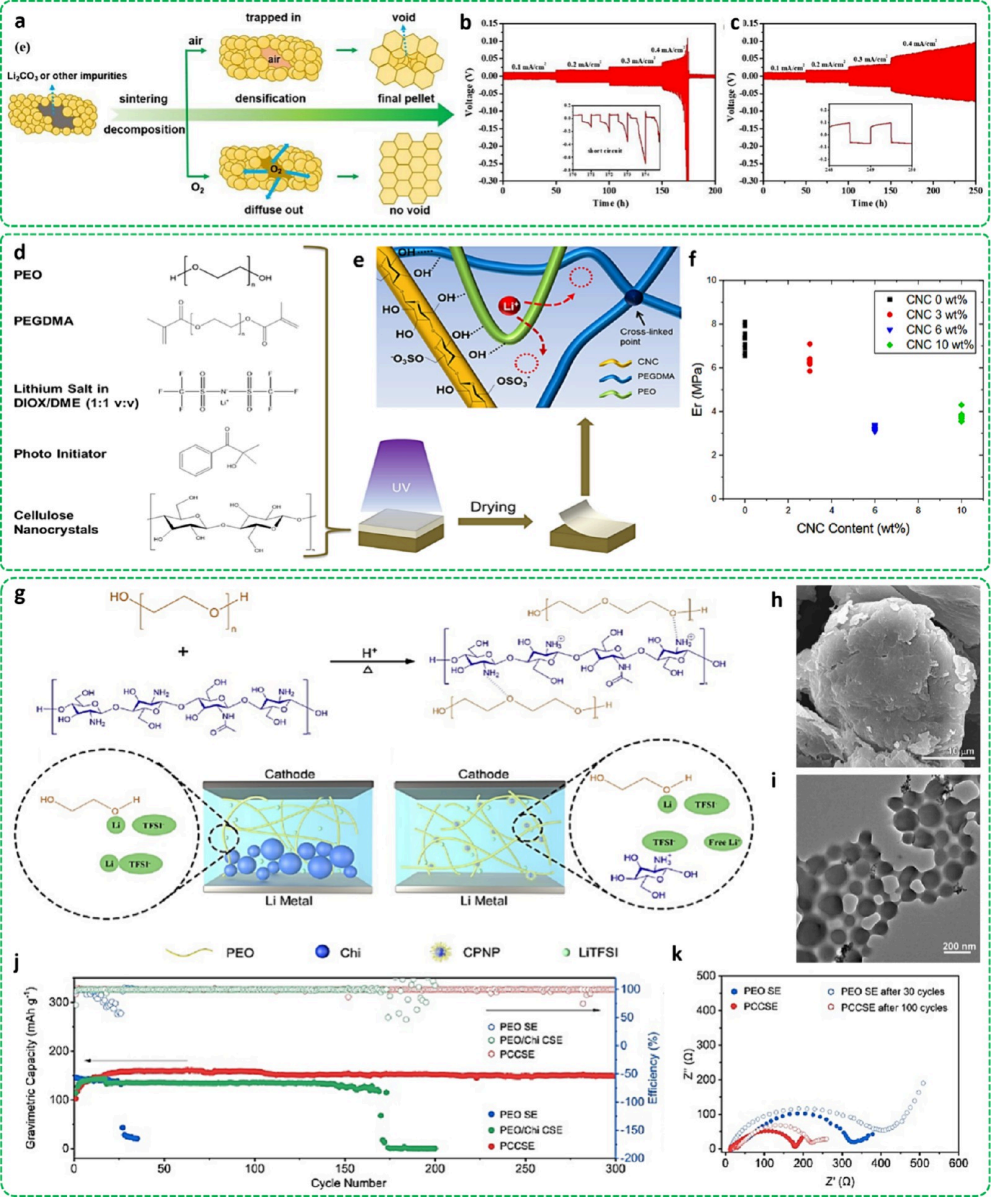
High-density garnet-type solid-state electrolyte: (a) illustrative of the sintering process and (b, c) the cyclic performance of LLZTO in air and LLZTO in O_2_ testified in Li|Li cells, respectively. Reproduced with permission from ref (52). Copyright [2020/American Chemical Society]. The cellulose presence and its role on PEO-based electrolytes: (d) Electrolyte precursors, (e) Mechanism of ion transportation in the designed electrolyte, and (f) Effect of cellulose contents on reduced elastic moduli of the composite electrolytes and median ionic conductivity, respectively. Reproduced with permission from ref (60). Copyright [2022/American Chemical Society]. Enhancing ionic conductivity of solid-state electrolyte by adding chitosan: (g) Synthesizing process and schematic of chitosan particle treatment in a developed electrolyte, (h) SEM and TEM of Chi-PEO nanoparticles, (i) Cycling performance of designed batteries at 0.5C, and (j) Rate performance of designed batteries at 0.2, 0.3, 0.5, 0.7, and 1 C in Li|LFP cells, and (k) Impedance spectra. Reproduced with permission from ref (62). Copyright [2023/Elsevier].

The Li_7_La_3_Zr_2_O_12_ (LLZO) garnet is considered to be a particularly attractive candidate for all-solid-state lithium batteries. A high-density LLZO pellet is preferred due to its ability to inhibit dendritic lithium growth and penetration. However, the traditional solid-state reaction used to prepare the LLZO electrolyte unavoidably results in the presence of pores. A significant number of pores can have a detrimental impact on both the ionic conductivity and density of the LLZO pellets. The research study investigated the formation of pores in the Li_6.4_La_3_Zr_1.4_Ta_0.6_O_12_ (LLZTO) and the fast oxygen-assisted sintering method (See Figure 4a). The properties of the LLZTO sintered in oxygen for only 1 h surpass those sintered in air across various physical parameters. For instance, the conductivity and Vickers hardness of the LLZTO increased to 6.13 × 10^–1^ mS·cm^–1^ and 9.84 GPa, with enhancements of 12.3% and 62.8%, respectively, even at a low precalcined temperature of 600 °C. As shown in Figure 4b,c, a Li||Li symmetric cell with the LLZTO sintered in oxygen demonstrates more stable and longer cycling at higher current density (0.4 mA·cm^–2^).^52^ Although garnet structures endow favorable electrochemical properties, their inflexible structure and high cost have implied challenges, motivating researchers to design polymeric nanocomposites decorated with garnet nanoparticles as filler components. In the polymeric configurations, garnet fillers disorder the matrix crystallization, facilitate ion pair dissociation, improve mechanical stability, and enhance the electrochemical efficacy. The beneficial role of garnet-type fillers in increasing ionic conductivity has been declared in myriad studies. For example, Lu et al.^53^ exhibited increasing the ion conductivity from 5.3 × 10^–1^ mS·cm^–1^ to 8.7 × 10^–1^ mS.cm^–1^ and enhancing the Li^+^ transference number from 0.38 to 0.48 via loading the LLZTO into a PEO-based SPE structure. The capacity retention was also enhanced from 88 to 92.5% after 200 cycles.

The enhancement in the ionic conductivity of SPEs has also been declared by incorporating talc,^18^ calcium carbonate,^54^ graphite,^55^ and others. Despite the numerous advantages of mineral fillers for generating versatile SPEs, the produced structures suffer from serious disadvantages, which includes a discussion of the implications of the findings for future research in this area. Mineral fillers often contain impurities, such as metal ions, which can contaminate the electrolyte and affect its electrochemical stability. In addition, the mineral particles could aggregate and form clusters within the polymer matrix, leading to nonuniform ion distribution and performance. In some cases, the filler particles are hygroscopic, resulting in degradation of the electrolyte properties. Many inorganic additives, such as heavy metals and halides, can be toxic to humans and the environment, limiting their use in electrolytes. Moreover, mineral additives can react with other components of the electrolyte and generate unwanted byproducts, and thereby, they result in electrolyte degradation and performance loss. The mining and processing of inorganic additives can have a negative impact on the environment, which is a particular concern for additives derived from rare or endangered materials. Furthermore, more endeavors are needed to explore ion migration theory in filler-loaded SPEs. When theory, multiscale modeling, and simulation are utilized in conjunction with experimental efforts, they can significantly contribute to closing current experimental and technological gaps. Additionally, they facilitate the prediction of path-independent properties and provide a deeper understanding of path-independent performance across various spatial and temporal scales.^56^ Accordingly, future attempts are required to address the challenges mentioned above by exploring new mineral fillers, modifying the surfaces of particles, synthesizing structures with controlled morphologies, developing multifunctional mineral galleries, and simulating the ion transport in these structures.

## 4 Polymer Electrolytes Embedded with Biobased Fillers

The current SPE-related technology aims to employ natural- or biobased fillers to combat the concerning issue of plastic contamination generated by synthetic galleries, as well as challenging obstacles of applying mineral structures. Biobased particulate fillers are abundant, environmentally friendly, sustainable, and cost-effective and can solve many downsides, including global warming, pollution, and price fluctuations, in tandem with other ecological and economic issues. The common biobased fillers utilized for boosting the electrochemical behavior of the SPEs could be classified based on their resources into natural polymers, gums, and biowastes, which are discussed in-depth in this section.

### 4.1 Natural Polymeric Fillers

Biopolymers are regarded as simple macromolecules generated or extracted from natural resources such as microbes or plants. There is a growing interest in replacing petroleum-based products using biopolymers as green and eco-friendly complex structures.^57^ Cellulose, chitosan, lignin, starch, and alginate are well-known and widely used biopolymers in various applications. Cellulose is a biodegradable, stable, and economical biopolymer known as a preferred material for generating battery components.^58^ The use of cellulose and its derivatives has been reported in several studies as key green additives for boosting the inherent characteristics of the SPEs. Based on the literature, cellulosic fillers could be a favorable choice for enhancing the ionic conductivity in polymer electrolytes. In this era, Samir et al.^59^ introduced rodlike natural cellulosic microcrystals into an SPE structure to enhance the electrochemical performance and mechanical strength of the provided composite. The storage modulus of these cellulosic nanocomposites exceeded that of unfilled polymer electrolytes more than 100 times. Even when compared to polymer electrolytes reinforced by TiO_2_, the storage modulus of the cellulosic nanocomposites still surpassed it by more than 50 times. The cellulosic nanocomposites maintained high conductivities across a wide range of filler loadings and reduced the internal resistance of the battery by more than 100 times.

To render a flexible energy source, a cellulose nanocrystal (CNC) reinforced electrolyte was tailored by decorating a PEO-based electrolyte with cellulose nanocrystal galleries. Figure 4d,e schematically displays all precursors and performance mechanisms of the designed SPE. By incorporating CNC into PEO and forming a cross-linked semi-interpenetrating polymer network, a robust polymer electrolyte was fabricated. The addition of CNCs inclined the PEO chain flexibility, facilitating Li^+^ transport. CNCs’ surface hydroxyl groups could interact with PEO ether oxygen moieties, disrupting crystalline PEO regions and promoting more Li^+^ carriers through forming hydrogen bonding interactions with lithium salt anions.

The incorporation of a modest amount of cellulose additive into the composite enhanced the mobility of the PEO segments, resulting in increased flexibility of the composite material. This softening effect is advantageous for shaping the electrolyte to meet the specific requirements of battery production. However, exceeding a 15 wt % proportion of CNCs led to a heightened rigidity of the composites, as well as less bending property (See Figure 4f). In comparison to PEO, the polymer electrolytes demonstrated proper thermal stability with enduring temperatures of up to 300 °C. Notably, when the CNC content constituted 10 wt % of the PEO fraction, the resultant flexible electrolyte exhibited equivalent ionic conductivity at 20 °C when compared to that of the control. Based on the data, it was observed that the ionic conductivity of the 10 wt.% CNC-loaded composite exhibited an approximate 30% increase across the relevant temperature range compared to the control sample.^60^ In the Tahir et al.^61^ investigation, waste cooking oil (WCO) served as the primary material for fabricating SPE films employing a solvent-free technique. The process commenced with pretreatment of WCO and its conversion into polyol via epoxidation and hydroxylation reactions. Subsequently, the WCO-derived polyol was compounded with diisocyanate, LiCF_3_SO_3_, and Carboxymethyl cellulose (CMC) to yield polyurethane SPE films. The addition of 15 wt % CMC increased ionic conductivity up to 1.19 × 10^–2^ mS.cm^–1^. The ionic conductivity supported with reduced crystalline peak intensity in XRD shows that the amorphous nature of SPE increased as more CMC was added. Furthermore, the tensile strength exhibited an incremental trend with the addition of CMC, culminating at 34.17 MPa with 10% CMC due to efficacious hydrogen bond interactions between CMC and polyurethane or salt. Nevertheless, a further increase in CMC content to 15% led to a reduction in tensile strength attributed to the agglomeration of CMC particles.

Chitosan is also a natural polysaccharide derived from chitin, which is a structural component of crustacean shells. As a biodegradable and sustainable filler, chitosan reinforces the mechanical strength, enhances the electrochemical stability, and boosts the ionic conductivity in SPE networks. Considering the advantages of chitosan, Huang et al.^62^ employed chitosan filler in an SPE structure, regarding the surface cations in its structure, the small size of the particles, and exceptional compatibility with polymers. The unique fillers were prepared through the reaction between PEO and chitosan at 85 °C, yielding Chitosan-PEO nanoparticles (CPNP) after a series of processes, including drying, cosolubilization, and centrifugation. A composite solid-state electrolyte (PCCSE) was then formulated by combining PEO, LiTFSI, and CPNP. The synthesizing procedure, chemical structure of CPNP, and its function in PEO-based CSEs are represented in Figure 4g. The obtained CPNP particles were remarkably finer than the original chitosan particles, retaining a minor amount of PEO on their surface. Consequently, the CPNP filler demonstrated exceptional dispersion in organic solvents, facilitating the fillers’ compatible interaction with the polymer matrix. The morphological structures of the synthesized nanoparticles are illustrated in Figure 4h,i. The original chitosan displayed a rough surface topography with dimensions around a few microns. However, as a result of the reaction between PEO and chitosan, CPNP exhibited a more uniform structure and smaller size. Notably, the CPNP size is intricately linked to the reaction temperature, as evidenced by the preparation of CPNP with diameters of approximately 500, 350, and 200 nm at 30, 60, and 85 °C, respectively.

In order to conduct a comprehensive assessment of the PCCSE performance in complete batteries, LFP||SSEs||Li full cells were constructed to evaluate their extended cycling performance at 0.5 and 50 °C, as depicted in Figure 4j. The discharge-specific capacity of LFP||PCCSE||Li measured approximately 159 mAh.g^–1^ after the initial activation and sustained the capacity of 149 mAh.g^–1^ after 300 cycles. The examinations also signified the Coulombic efficiency and capacity retention, reaching up to 99 and 94%, respectively, after 300 cycles. Conversely, PEO solid-state lithium batteries (PEOSLBs) exhibited a considerably shorter cycling life, lasting only 27 cycles at 0.5 C, with an average discharge-specific capacity of 140 mAh.g^–1^, markedly lower than PCSLBs. Furthermore, batteries featuring a PEO/Chitosan CSE demonstrated a lifespan of 169 cycles with a reduced discharge-specific capacity of 134 mAh·g^–1^ and an escalating polarization voltage. To understand the role of PCSLBs on the electrolyte performance, the EIS of batteries before and after cycles was examined (See Figure 4k). As a result, the PCSLBs have lower original resistances (179 Ω) compared with PEOSLBs (325 Ω). After cycling, PCSLBs maintained a relatively stable impedance of 222 Ω after 100 cycles, while PEOSLBs showed impedance of 399 Ω after 30 cycles. This examination suggested that adding CPNP could provide proper compatibility between electrolytes and electrodes, resulting in lower resistance during charging and discharging and higher ionic conductivity, ultimately giving PCSLBs longer cycling life. The electrochemical performance of PCCSE exhibited significant improvement, primarily attributed to the introduction of cations in CPNP, which featured an abundance of -NH_3_^+^ groups. The -NH_3_^+^ groups generated during the CPNP protonation process could interact with TFSI in the solid-state electrolytes (SSEs), leading to enhanced dissociation of LiTFSI and increased release of free Li^+^ ions, consequently further augmenting the ionic conductivity of PCCSE. Moreover, CPNP demonstrated a smaller size and superior dispersion than the untreated chitosan, with each chitosan nanoparticle tightly enveloped by PEO, thereby providing a larger interface area and more efficient transport paths for Li^+^.

Lignin has also been known as another readily available, underutilized biopolymer with promising characteristics that are favorable for boosting the performance of various battery components, including electrodes, binders, and electrolytes. In an attempt performed by Liu et al.,^63^ lignin was incorporated into a PEO-based SPE as an additive. The results showed that shifting of the melting point to a lower temperature and the formation of wider peaks in XRD spectra corroborated a reduction in the formation of crystalline phases. By decreasing the crystalline phases, faster internal modes were attained in PEO polymer chains; thereby, superior segmental movements occurred. Therefore, the conductivity was inclined from 3.4 × 10^–8^ to 10^–2^ mS·cm^–1^ via embedding 0.1 wt % lignin filler. Although numerous studies have evaluated the use of various biopolymers as polymer host matrices, poor attention has been paid to exploring their roles as green biofillers. In future investigations, it might be possible to employ biopolymers in various configurations with different compositions to enrich the SPE structures.

### 4.2 Protein-Based fillers

**Figure 5:**
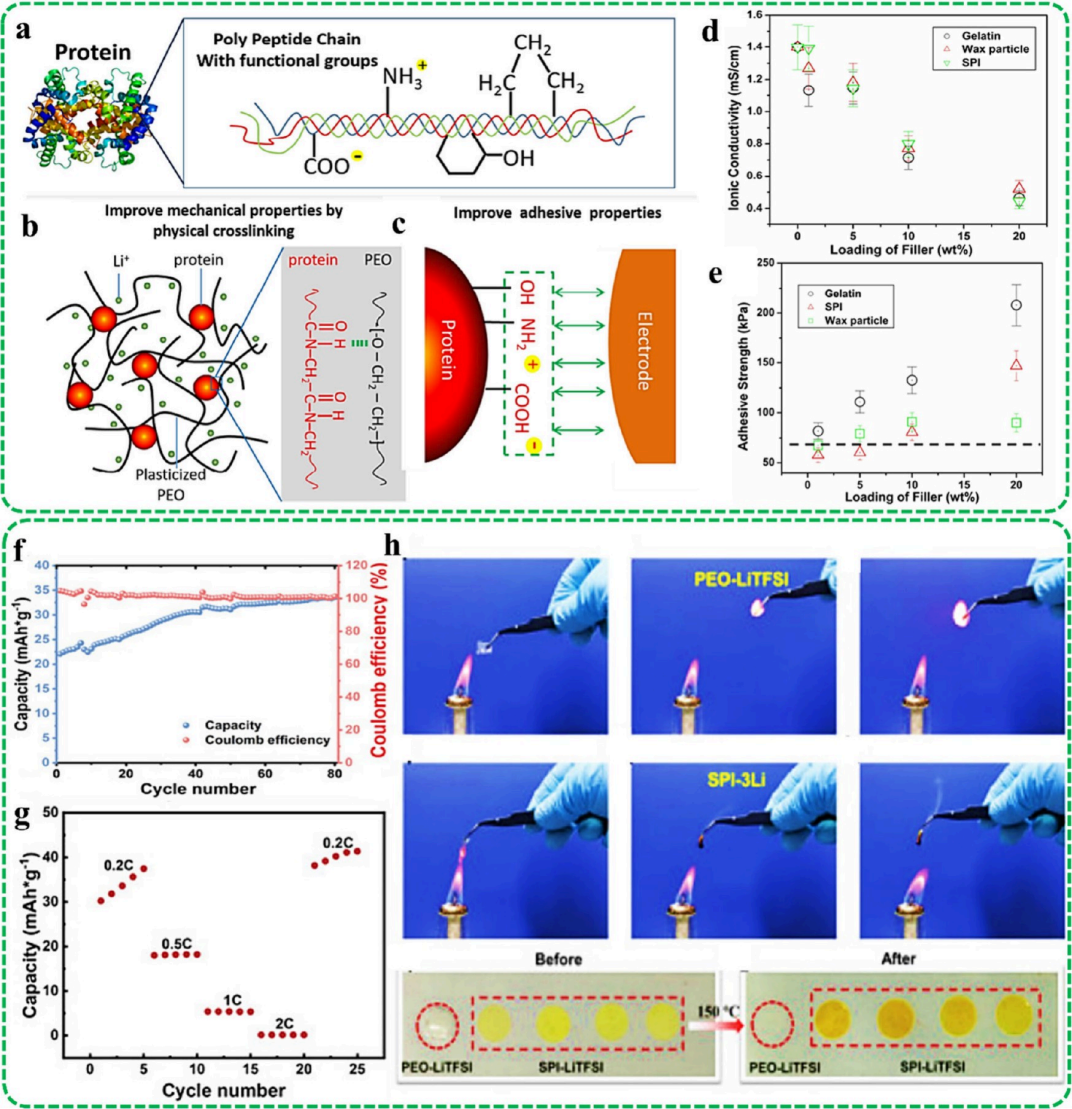
Animal-derived gelatin, Wax, and SPI biofillers for integrating a PEO-based SPE: (a) The functional groups of the protein fillers, (b) The mechanism of ion conductivity in an SPE structure filled with protein biofiller, (c) The effect of protein on enhancing the adhesive strength with the substrate, (d) Ionic conductivity versus filler volume, and (e) Adhesive strength versus filler loading concentration. Reproduced with permission from ref (64). Copyright [2016/Elsevier]. Characteristics of a SPE architecture embedded with SPI filler: (f) The specific discharge capacity of the generated cell at 0.2 C at 60 °C, (g) The specific discharge capacity of the cell at various 0.2, 0.5, 1, 4, and 0.2 C rates in Li|LFP cells, and (h) Flame retardancy of the filler-free and filler-loaded membranes when contacting the fire. Reproduced from ref (65). Available under a CC-BY 4.0 license. Copyright [2022] Gu et al.

Proteins are also classified as naturally abundant materials that reinforce polymeric structures by providing various benefits. As an essential and classic biomaterial, proteins serve appropriate physical, chemical, and biological activities, thereby reaping remarkable attention for the development of next-generation high-energy rechargeable batteries. In this era, Wang et al.^64^ compared the impact of animal-derived gelatin, Wax, and soy protein isolate (SPI) as protein biofillers on mechanical strength and electrochemical efficiency of a PEO-based electrolyte. Regarding various amino acid types in the protein structures, these networks comprise diverse functional groups, including −COOH, -NH_2_, −OH, etc. (See Figure 5a). Accordingly, such additives could form homogeneous particles in both liquid- and solid-based electrolytes. In the SPE architectures, proteins create strong interactions with PRO polymer chains through peptide bonds, as well as functional groups, forming physical cross-linking (See Figure 5b). The observed linkages provide remarkable mechanical strength with appropriate elasticity. As can be seen in Figure 5c, strong adhesion could be provided with various substrates, such as electrodes, enhancing both electrochemical and mechanical properties. Figure 5d exhibits the ion conductivity of the generated SPEs versus the filler loading. Based on the obtained data, the ionic conductivity was enhanced via loading more filler content. In addition, the filler loading displayed a higher effect than the filler type on boosting the ionic conductivity. The adhesive strength of the produced electrolytes with the surface of the electrodes is illustrated in Figure 5e. The attained data exhibited the increment of adhesive strength by raising the gelatin and SPI content in the electrolyte, assigning to the strong interactions between the particulate fillers and the substrate, whereas inclining the Wax filler caused a reduction in the adhesive strength.

In another attempt, Gu et al.^65^ embedded SPI as particulate filler into an SPE structure comprising LiTFSI as a lithium salt. Based on the obtained data, a high ionic conductivity of 3.3 × 10^–1^ mS.cm^–1^ was approached, resulting from the presence of this filler in the polymeric network. According to the analysis, the SPI is constructed from 18 different amino acids, which are linked by covalent bonds. Frequent positively charged amino acids, including arginine, lysine, and histidine in the SPI structure, can feasibly trap and immobilize CF_3_SO_3_^–^ anions, accelerating the Li^+^ ion motion in the designed SPE. The designed electrolyte could maintain the specific discharge capacity of 32.6 mAh.g^–1^ and a Coulombic efficiency of about 100% after cycling, corroborating proper electrochemical stability (See Figure 5f). The function of the cell at various current densities of 0.2, 0.5, 1, 4, and 0.2 C was also analyzed. The results figured out various discharge capacities of 33.6, 18.1, 5.3, 0.15 mAh·g^–1^ at 0.2, 0.5, 1, 4 C and prompt recovery to 39.1 mAh.g^–1^ after returning to 0.2 C (See Figure 5g). Moreover, the generated film exhibited proper mechanical stability, even with increasing atmospheric humidity, which could be attributed to the easy water adsorption of the soy protein isolate element. The flame retardancy of the integrated electrolyte was also examined by considering the filler-free sample as a reference. The investigation exhibited immediate burning of the reference SPE in contact with fire. Meanwhile, the filler-loaded sample was distorted without causing fire and represented a proper flame retardancy, which could be linked to the interaction between the embedded filler and the lithium salt (See Figure 5h).

Overall, protein-based electrolyte additives are able to enhance the Li^+^ ion transference number by blocking the anions and immobilizing the Li anode surface, inhibiting the growth of Li dendrites, and repairing the solid electrolyte interface. This could lead to a homogeneous distribution of the Li^+^ ions, as well as increasing the cycling life. Despite the influential role of protein-based fillers in escalating efficiencies of the electrolytes, more attempts in this era are dedicated to exploring the impact of protein additives on liquid electrolytes.^66^ Future studies on embedding protein fillers into SPE architectures are therefore recommended.

### 4.3 Biowaste Fillers

**Figure 6:**
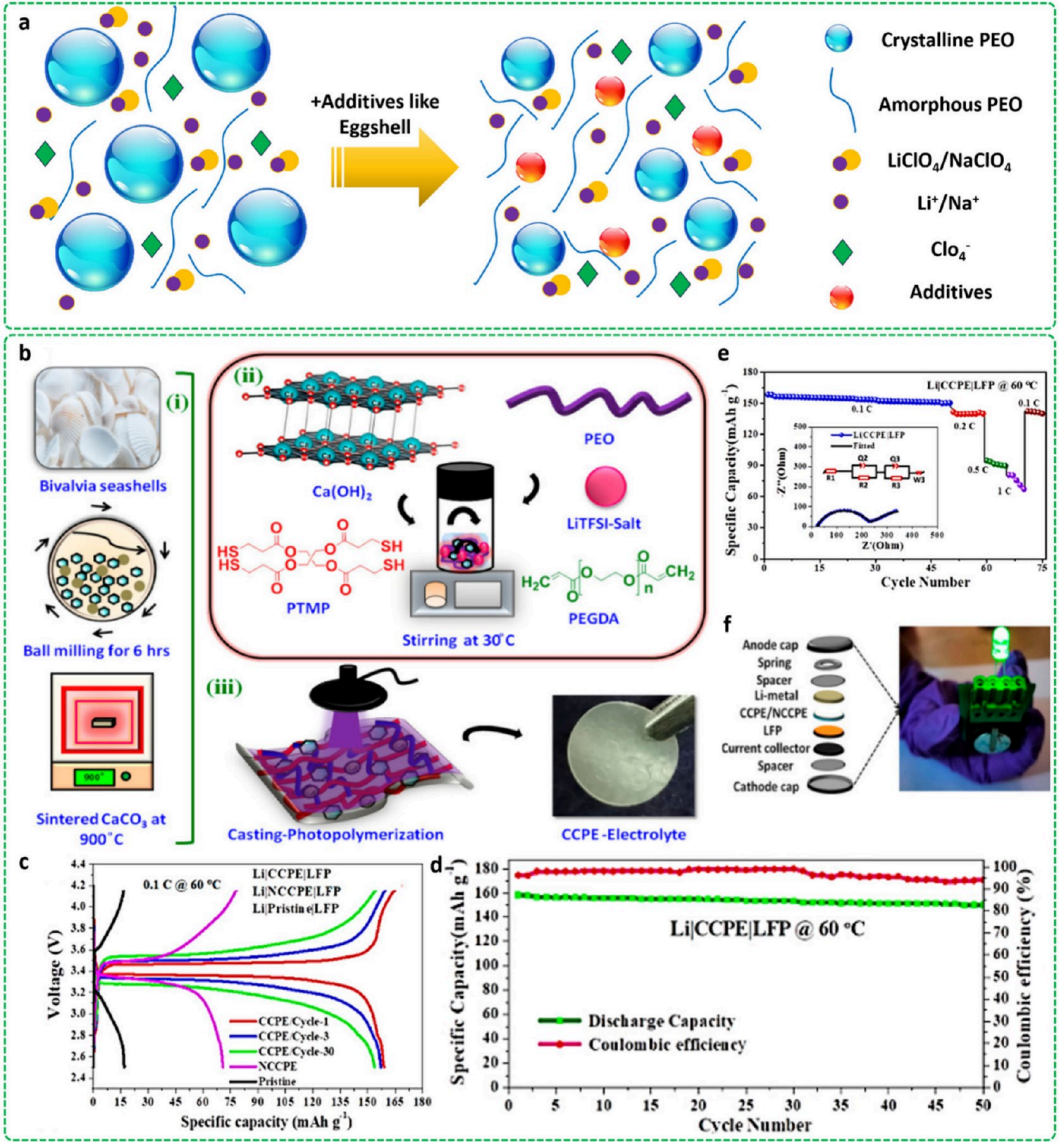
Addition of eggshell waste as filler into a PEO-based SPE: (a) Schematic illustration of the ion transfer mechanism in both filler-free and filler-loaded membranes. The role of Seashell waste in enhancing the ionic conductivity: (b) Schematic illustration of the synthesis procedure, (c) Full cell charge/discharge performance of the designed SPEs, (d) Specific capacity and Coulombic efficiency of the CCPE structure, (e) Specific capacity of the Li|CCPE|LFP full cell in various C rates, and (f) Using the optimized network for turning on a green LED in Li|LiFePO_4_ cells. Reproduced with permission from ref (68). Copyright [2023/American Chemical Society].

A significant rise in the population has caused an increasing amount of goods and, therefore, biowastes every year. As an example, food industries dispose of a large number of eggs and seashells in landfills, posing a health threat to the public by attracting worms and rats. Accordingly, the use of biowaste materials has reaped the attention of researchers to generate new age devices. Some attempts have also been made to use biowaste fillers to enhance the electrochemical performance of SPE structures. As an example, Figure 6a shows a schematic illustration of the role of eggshell-based fillers in enhancing ionic conductivity. To conquer the ionic conductivity of a PEO-based SPE, Xu et al.^67^ incorporated waste eggshell-derived microfillers. According to the conducted study, chicken eggshell was washed by using deionized water to diminish impurities and dried at 60 °C for 24h. Afterward, the ball-milling and calcination procedures were employed to obtain fine particulate eggshell fillers. The obtained particles were mainly CaO, which could form close contact with the PEO polymer chains, leading to a reduction in crystallinity, as broader and weaker peaks in the XRD spectra confirmed it. Based on this, the presence of fillers in a composite polymer electrolyte caused an approximately 4.5-fold increase in ionic conductivity in comparison to the polymer reference electrolyte. The addition of 7 wt.% filler caused an enhancement in the Li ionic conductivity up to 6.39 × 10^–2^ mS·cm^–1^, while the highest ionic conductivity of 4.9 × 10^–2^ mS.cm^–1^ was obtained for the Na conduction through embedding 5 wt.% fillers. The observed improvement in the ionic conduction of the SPEs could be linked to the destruction of the crystalline regions as well as prompt absorption and desorption of Li and Na ions by the filler particles. Notably, the designed cell represented a stable cycling behavior through delivering a reversible discharge capacity of 142.8 mAh.g^–1^ after 200 cycles.

Swathi et al.^68^ extracted calcium hydroxide from waste seashells and embedded the provided bioparticles as filler into a PEO-based SPE structure. The photopolymerization technique was employed in the fabrication procedure to cross-link monomers. Figure 6b presents a schematic illustration of the fabrication procedure used for preparing the SPE structure. The loaded filler acted as a cross-linking center, resulting in an increment in the amorphous regions. Additionally, the obtained calcium hydroxide showed a porous hexagonal structure, which could be smoothly dissociated with lithium salt ion pairs, as well as the PEO polymer chains. The modified structure also figured out a proper discharge capacity of 160 mAh.g^–1^ with a high Coulombic efficiency of 95% after 50 cycles.

Moreover, the cross-linking monomers caused the formation of a rigid polymeric network with appropriate mechanical properties. To compare the synergetic effect of the calcium hydroxide additive and cross-linking procedure on enhancing the electrochemical performance, three samples of pristine SPE, non-cross-linked composite (NCCPE), and cross-linked composite (CCPE) were generated. Figure 6c illustrates the charging and discharging profiles of the full cells, indicating discharge capacities of 15, 70, and 160 mAh.g^–1^ at 0.1 C for the pristine membrane, NCCPE, and CCPE, respectively. The introduction of porous Ca(OH)_2_ filler provided superior surface area, favoring charge–discharge capacity and Li-ion transferring. The cross-linking treatment also improves ion mobility by creating a functionally aligned cross-linked structure. The Coulombic efficiency performance of the designed membranes is also depicted in Figure 6d, declaring a high Coulombic efficiency of 95% at 0.1 C. Figure 6e also exhibits the cycling performance of the Li|CCPE|LFP full cell after subjecting it to various C rates. Furthermore, the Li|CCPE|LFP full cell could turn on a green LED after 50 cycles at 0.1 C (Figure 6f).

Natural or biowaste materials are readily available, making them a sustainable and cost-effective option for filler materials. As these materials are biodegradable, they can diminish environmental concerns associated with the disposal of spent electrolytes. The properties of such fillers can be tailored by varying the source, processing conditions, and surface modifications, allowing for the optimization of electrolyte performance. Although natural or biowaste fillers have exhibited several superiorities, few studies have engaged with analyzing these fillers due to some challenging concerns, which should be addressed in the future. For example, these materials can contain impurities and exhibit batch-to-batch variations, which may affect the consistency and performance of the electrolyte. Some fillers are hygroscopic and can absorb moisture from the environment, potentially leading to decreased ionic conductivity and electrolyte degradation. These fillers may not be compatible with all polymer matrices, requiring proper surface treatments or compatibilizers to ensure good adhesion and dispersion. Incorporating natural or biowaste fillers into polymer electrolytes can be challenging due to their tendency to agglomerate or form clusters, which can hinder ion transport. Furthermore, the cost and scalability of natural- or biowaste-derived fillers need to be carefully considered for large-scale production of SPEs.

## 5 Integration of Polymer Electrolytes Via Loading Hybrid Mineral and Biobased Fillers

**Figure 7:**
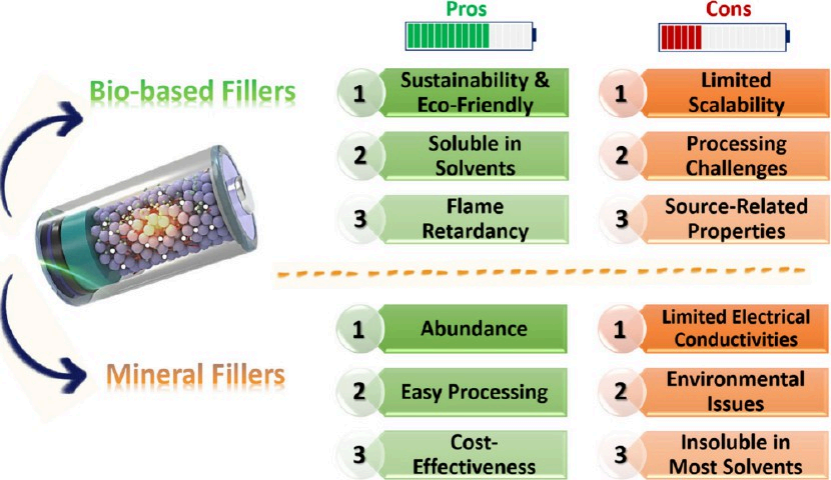
Schematic design showing the advantages and disadvantages of biobased and mineral fillers.

Providing organic and inorganic fillers from bio- and natural-based resources has shown several advantages and disadvantages, as listed in Figure 7. Accordingly, mineral fillers mainly serve finer grains than biobased particles due to tighter packing. They are also readily available worldwide and could be provided at lower cost. Meanwhile, biobased fillers could be easily dissolved in water or other organic solvents. Additionally, they lead to the generation of lighter composites because of their lower density. Biobased structures are also derived from sustainable and renewable resources, making them more eco-friendly than mineral structures. To inhibit the reported cons and maximize the benefits of natural structures, the use of combined fillers could be a favorable strategy.

This study section explores the potential synergistic effects of incorporating biobased and mineral fillers into SPE structures. It is anticipated that the combined presence of these fillers leads to a more optimized electrolyte assembly, offering a wide range of distinct advantages, including enhancing ionic conductivity, amplifying mechanical properties, reducing flammability, and enhancing high-cost efficiency. In this regard, Zhou et al.^69^ employed the PVDF matrix due to its proper mechanical strength and low price, which integrated with SPI and MMT to enhance the electrochemical properties. According to the results, the SEM image of the PVDF/LiTFSI film depicted a smooth surface without obvious pores. However, after the addition of SPI and MMT, pores became visible among some spherical structures in the film.

The pores displayed regular and orderly diameters with no evidence of serious phase separation, indicating good compatibility among SPI, MMT, and the polymer matrix. The corroborated compatibility could be attributed to the formation of the N–H–O hydrogen bond by the amine group in SPI and the hydroxyl group in MMT. This strong interaction led to better synergy between SPI and MMT, enhancing the Li-ion migration and conductivity of the material. Based on the findings, it was observed that the tensile strength and deformability of the polymer electrolyte decreased significantly after adding SPI, assigned to the reduction of the crystalline area within the polymer network. However, upon the addition of MMT to the PVDF/SPI/LiTFSI membrane, there was a notable improvement in the tensile strength of the polymer electrolyte. This enhancement could be attributed to the strong interaction between MMT and the polymer segment.

Consequently, the overall performance of the polymer electrolyte was considered satisfactory when both SPI and MMT were added simultaneously. The PVDF/SPI/MMT membrane exhibited an ionic conductivity of 2.56 × 10^–1^ mS.cm^–1^ at room temperature, with a high Li^+^ ion transference number of 0.77. The membrane also demonstrated an inclusive electrochemical stability window of 5.1 V. The performance of the Li-ion polymer battery was evaluated across 100 cycles, discharging at a current of 0.5 C within a potential range of 2.75 to 4.00 V. A gradual reduction in discharge capacity was noted with an increasing number of cycles, ascribed to deteriorating interface between the polymer electrolyte and the electrode, as well as the development of an interface passivation layer, which results in the reduction of active material and overall battery capacity. The specific discharge capacity of the original polymer electrolyte decreased from 103 to 30 mAh.g^–1^ over 100 cycles. Conversely, with the addition of SPI and MMT, the specific discharge capacity fell from 100 to 55 mAh.g^–1^. This indicated that the incorporation of fillers improved the interface compatibility between the electrolyte and the electrode, consequently enhancing the battery’s cycle performance.

**Figure 8:**
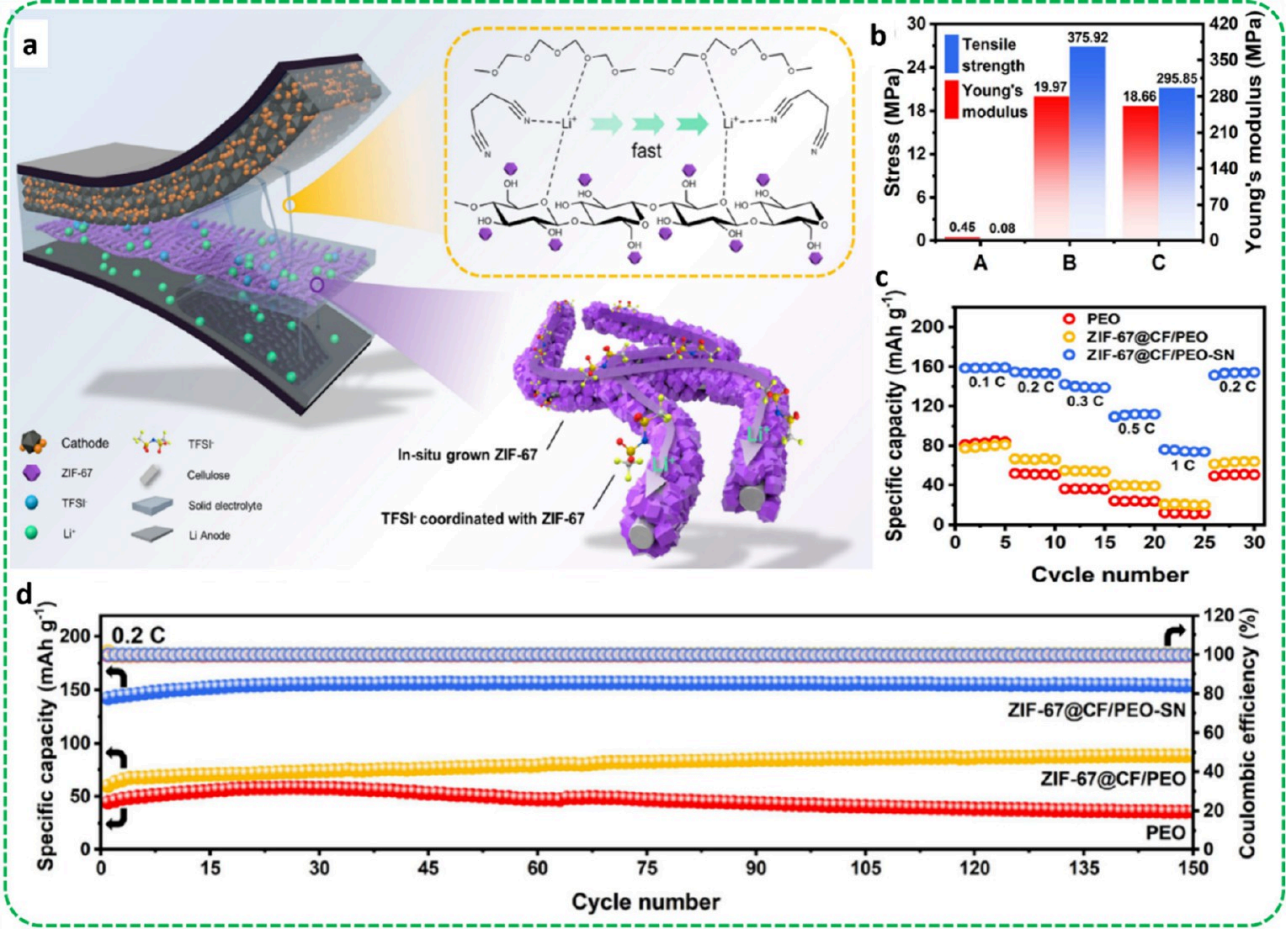
ZIFs/Cellulose framework effect on reinforcement of polymeric electrolyte: (a) Schematic of structures, (b) The stress- Young’s modulus of different samples, (c) Specific capacity diagram of various designed cells at 30 °C at several rates, and (d) charging–discharging cycling performance of various designed cells in Li|LFP cells. Reproduced with permission from ref (70). Copyright [2024/American Chemical Society].

In another study, Song et al.^70^ attempted to represent a reinforced composite electrolyte by applying a cellulose and zeolitic imidazolate framework (ZIF) combination in a PEO-based network (See Figure 8a). According to the theory of this study, cellulose enhances the mechanical properties and thermal stability of composite polymer electrolytes without reducing the ion conductivity. Metal–organic frameworks (MOFs) are also crystalline porous materials formed through the self-assembly of organic ligands with metal ions, possessing high porosity and adjustable pore size. Tensile strength, along with Young’s modulus of samples, are exhibited in Figure 8b. The PEO matrix had a tensile strength of only 0.45 MPa and a Young’s modulus of 0.08 MPa. By incorporation of high-modulus 3D ZIF-67@CF, the mechanical properties of ZIF-67@CF/PEO and ZIF-67@CF/PEO-SN were significantly enhanced. Young’s modulus also showed a dramatic improvement, reaching 375.92 and 295.85 MPa, which are 4700 and 3700 times higher than that of the PEO matrix. Also, Young’s modulus and tensile strength of ZIF@CF/PEO-SN were slightly lower compared to ZIF@CF/PEO, likely due to the presence of a plasticizer in the structure. The lower tensile strength of ZIF-67@CF compared to CF is due to the in situ growth of ZIF-67, disrupting the hydrogen bonding of the cellulose part and reducing the tensile strength. According to Figure 8c, in LFP/(ZIF-67@CF/PEO)/Li cells, specific capacities during discharging were 82.0, 70.0, 40.0, 30.0, and 20.0 mAh.g^–1^, showing only marginal differences at low discharge rates due to similar ionic conductivities. LFP/(ZIF-67@CF/PEOSN)/Li cells displayed excellent discharging capacities and stability. The combination of ZIF-67@CF and a succinonitrile (SN) plasticizer significantly enhanced cycling stability and capacity. In cycling tests at 0.2 C and 30 °C, the LFP/(ZIF-67@CF/PEO-SN)/Li cell showed an initial discharging capacity of 142.3 mAh.g^–1^, increasing to 156.3 mAh.g^–1^ over 150 cycles with a retention rate of 99% and Coulombic efficiency of 99.5% (See Figure 8d). This outperformed the LFP/PEO/Li cell, which only reached 44.6 mAh.g^–1^ and retained 60% of its capacity after 150 cycles. The LFP/(ZIF-67@CF/PEO-SN)/Li cell also demonstrated outstanding cycling stability, retaining 83% capacity after 300 cycles at 1 C at 30 °C.

In summary, biobased fillers contributed to improving ionic conductivity by establishing a network of interconnected pores, facilitating more facile movement of Li^+^ ions within the electrolyte. Additionally, inorganic mineral fillers served to enhance the ionic conductivity through the provision of a more amorphous electrolyte structure. Also, incorporating biobased fillers fortified the mechanical properties of polymeric electrolytes owing to their inherent strength and rigidity, while inorganic fillers conferred greater rigidity and durability to the electrolyte structure, further improving its mechanical properties. Besides, biobased fillers inherently reduce the flammability of polymeric electrolytes due to their nonflammability. Moreover, the integration of inorganic mineral fillers imparts a more thermally stable structure to the electrolyte, thereby reducing its flammability.

Furthermore, biobased fillers present a cost-effective alternative to inorganic fillers, thereby contributing to an overall reduction in the cost of SPEs. Some of the important challenges of integrating biobased fillers with inorganic fillers in polymeric electrolytes for Li-ion batteries are reduced stability, processing challenges, and compatibility. Biobased fillers might show a lower stability than inorganic fillers, especially in the presence of moisture or high temperatures. Inorganic mineral fillers might also face stability issues in certain environments. Also, integrating biobased fillers with inorganic fillers could pose challenges due to poor intermixing, leading to difficulties in electrolyte formation and device performance. Finally, biobased fillers might not be universally compatible with all inorganic fillers, leading to potential problems in electrolyte formation and device performance. So briefly, while combining biobased fillers with inorganic mineral grains in SPEs can offer various advantages, there are also some disadvantages to consider, such as reduced stability and processing challenges. These factors should be carefully considered when designing and manufacturing Li-ion batteries.

## 6 Conclusion and Future Remarks

This overview has revealed a significant body of research dedicated to exploring the application of additives to enhance the electrolyte properties. A detailed examination of the merits and demerits of each additive type was presented. The study’s primary finding underscores the potential of an enhanced future for polymer electrolytes in solid-state batteries due to the synergistic and optimized utilization of bioadditives and mineral additives as natural organic and inorganic fillers.

Overall, all-solid-state polymeric electrolytes have been documented as a strong alternative to conventional liquid electrolytes in Li-ion batteries. Such structures are crucial for modern batteries, especially for electric vehicles and portable electronics. This promising candidate is characterized by its reduced flammability, which mitigates the risk of fire and explosion. Also, polymeric electrolytes can accommodate a higher concentration of lithium ions, hence, enabling the creation of batteries with greater energy density. Notably, these electrolytes exhibit operational viability across a wider temperature range, rendering them suitable for deployment under extreme environmental conditions. Their relative cost-effectiveness, when compared with traditional liquid electrolytes, further enhances their appeal. Augmenting the performance of polymeric electrolytes can be achieved through the incorporation of green filler materials derived from mineral and natural resources. Mineral fillers (such as mineral oxides, clay flakes, zeolite, garnet, talc, etc.) mainly contribute to improved ionic conductivity and mechanical properties, while biofillers (like natural polymers, proteins, and biowastes) enhance biodegradability and sustainability. As ongoing research endeavors continue to refine the performance of polymeric electrolytes, these alternatives are expected to advance prevalence in commercial battery applications. Advancements in polymeric electrolytes are estimated to stem from the development of new high-conductivity polymers, composite electrolytes, and inorganic and biofillers. Emerging polymers featuring elevated dielectric constants and reduced glass transition temperatures are under development to bolster the ion conductivity. Simultaneously, the refinement of mineral and biofillers reflects progress aimed at enhancing their properties. However, their challenging production process necessitates more skills and attention. Also, the properties and performance of fillers can be integrated by modifying their morphology, structure, and arrangement. This includes producing fillers in fibers, layers, blade shapes, and so on. On the other hand, developing composite electrolytes harnessing diverse material advantages is also underway to engender optimal performance. Besides, as an emerging technique, employing the composition of biobased and mineral fillers could be promising toward combatting the gaps and approaching favorable outcomes. These advancements are hoped to precipitate the realization of polymeric electrolytes endowed with heightened ion conductivity, enhanced mechanical properties, and bolstered safety measures. Consequently, polymeric electrolytes are poised to emerge as a compelling choice for integration into Li-ion batteries, potentially yielding batteries with amplified energy density, prolonged life cycles, and reduced production costs.

**Figure 9:**
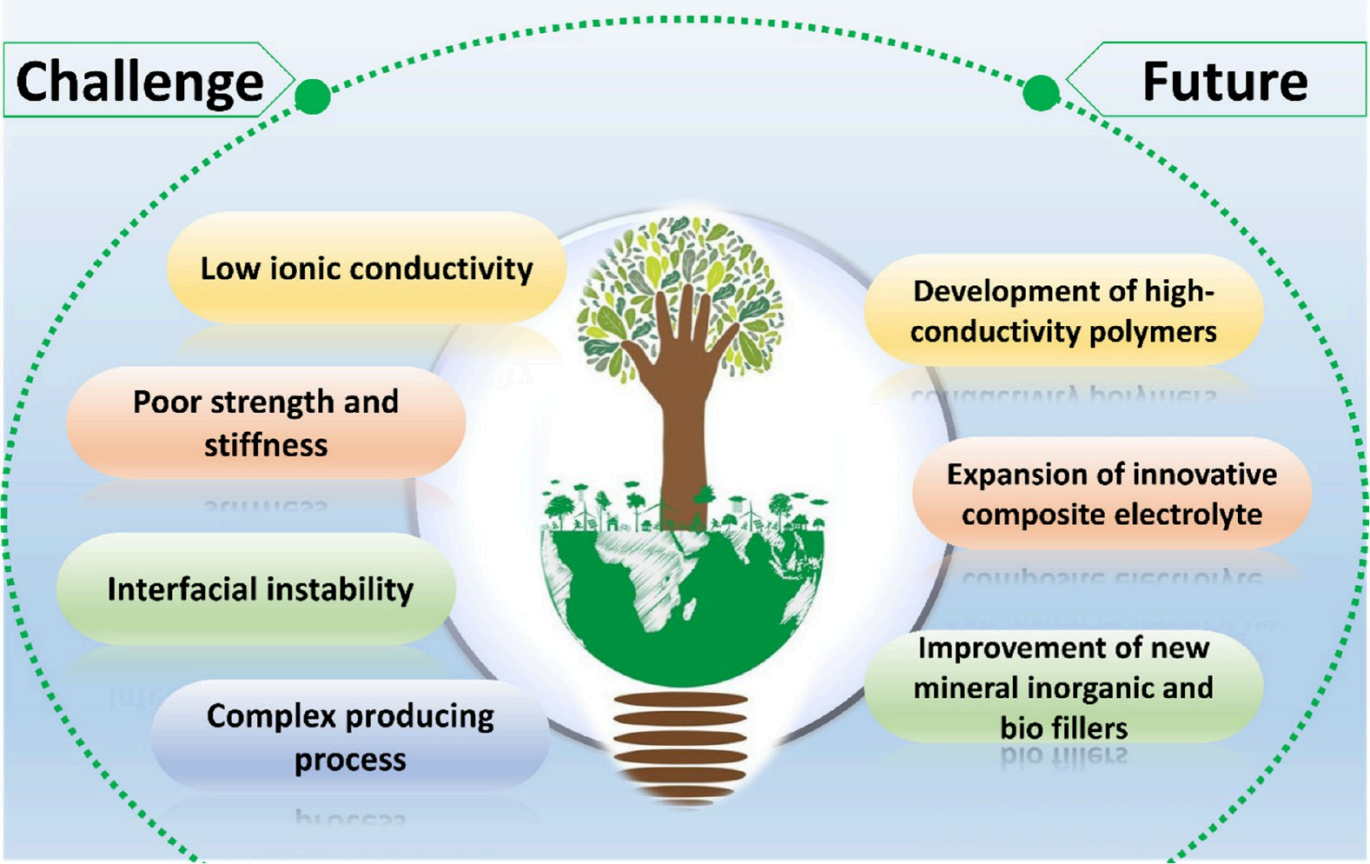
Perspective view and gaps of new generation Li-ion batteries incorporated with mineral and biobased fillers.

Despite the numerous advantages of polymeric electrolytes in Li-ion batteries, critical gaps and challenges require attention before widespread commercialization becomes viable, as summarized in Figure 9. Primarily, the ionic conductivity of polymeric electrolytes continues to lag behind that of liquid electrolytes, thereby restricting the power and energy density of batteries employing polymeric electrolytes. Polymeric electrolytes exhibit inferior strength and stiffness compared to their liquid counterparts, rendering them more susceptible to damage during battery assembly and usage. Furthermore, polymeric electrolytes are prone to instability when interfacing with the lithium metal anode, forming a solid electrolyte interphase layer that obstructs the flow of lithium ions, ultimately leading to battery failure. Finally, the manufacturing costs of polymeric electrolytes surpass those of liquid electrolytes, diminishing their appeal for cost-sensitive applications. In addition to these general challenges, utilizing inorganic and biofillers in polymeric electrolytes presents specific hurdles. For instance, inorganic fillers often pose challenges in achieving uniform dispersion within polymeric electrolytes, thus promoting the formation of agglomerates that impede the flow of lithium ions. Moreover, inorganic fillers can display instability in the presence of moisture and other impurities. As for biofillers, they are typically less stable than their inorganic counterparts and can present challenges in achieving uniform dispersion within polymeric electrolytes. Furthermore, biofillers can be susceptible to degradation by microorganisms.

Nevertheless, ongoing research endeavors seek to address the above-mentioned gaps and challenges associated with utilizing polymeric electrolytes in Li-ion batteries. Novel polymers, fillers, and processing techniques are under development to enhance performance and mitigate the costs associated with polymeric electrolytes. With continued efforts, it is expected that polymeric electrolytes will gain increasing prevalence in commercial Li-ion batteries.

## Supporting Information Available

Depicting the bibliographic map of Scopus-indexed papers linked to the Solid-state-electrolyte and Lithium-ion battery keywords, alongside the number of publications dedicated to analyzing the filler-loaded membranes applicable in lithium-ion batteries (PDF)
