# Supplementary material for: Advancements in Battery Materials: Bio-Based and Mineral Fillers for Next-Generation Solid Polymer Electrolytes
Source: ACS Appl Mater Interfaces. 2024 Nov 7;16(46):63089–108. doi: 10.1021/acsami.4c11214 (PMC11583125; doi:10.1021/acsami.4c11214)
Supplement: Supplementary file 1 — am4c11214_si_001.pdf [file am4c11214_si_001.pdf]

Supporting Information

Advancements in Battery Materials: Bio-Based and Mineral Fillers for Next-Generation Solid Polymer Electrolytes

Tayyab Subhani *[1], Sanaz Khademolqorani [2], Seyedeh Nooshin Banitaba [2], Mohamed Ramadan [1, 3], Abdul Khaliq [1], Imran Ali Chaudhry [1], Ahmed I. Osman *[4]

[1] *College of Engineering, University of Ha'il, P.O. Box 2440, Hail 81481, Saudi Arabia*

[2] *Emerald Experts laboratory, Isfahan Science and Technology Town, Isfahan 84156-83111, Iran*

[3] *Central Metallurgical Research and Development Institute (CMRDI), P.O. Box 87, Helwan 11421, Egypt*

[4] *School of Chemistry and Chemical Engineering, Queen's University Belfast, Belfast BT9 5AG, Northern Ireland, UK*

Correspondence to: (*Tayyab Subhani (ta.subhani@uoh.edu.sa) and *Ahmed I. Osman (aosmanahmed01@qub.ac.uk))

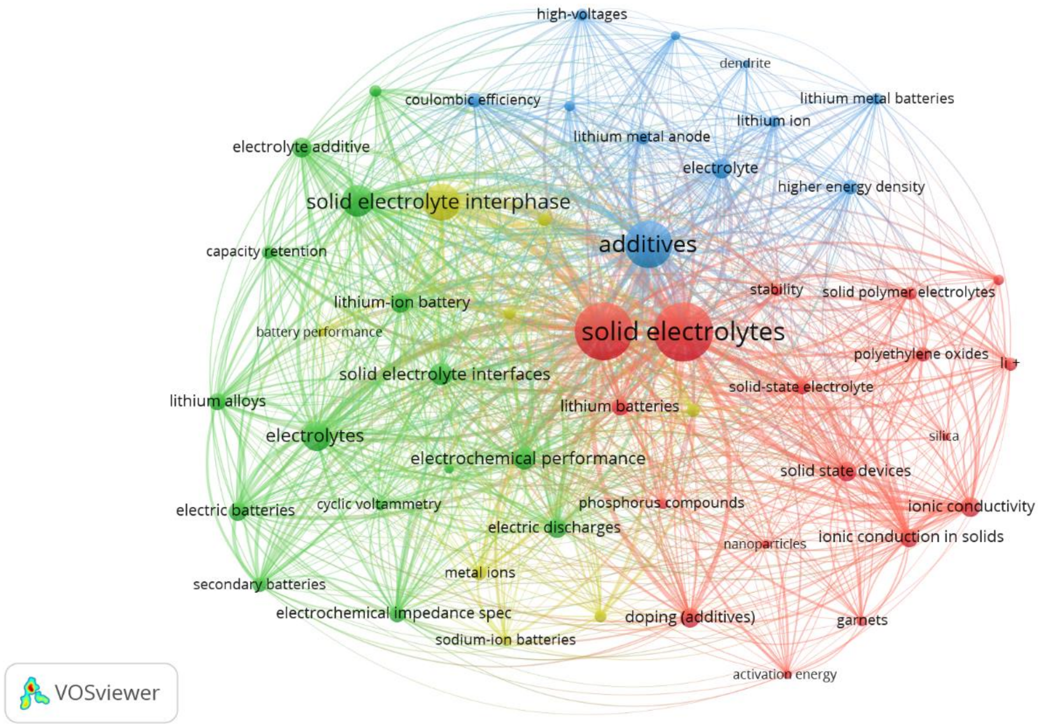

Fig. S1 Map of bibliographic coupling of Scopus-indexed papers related to the keywords of Solid-state-electrolyte and Lithium-ion battery

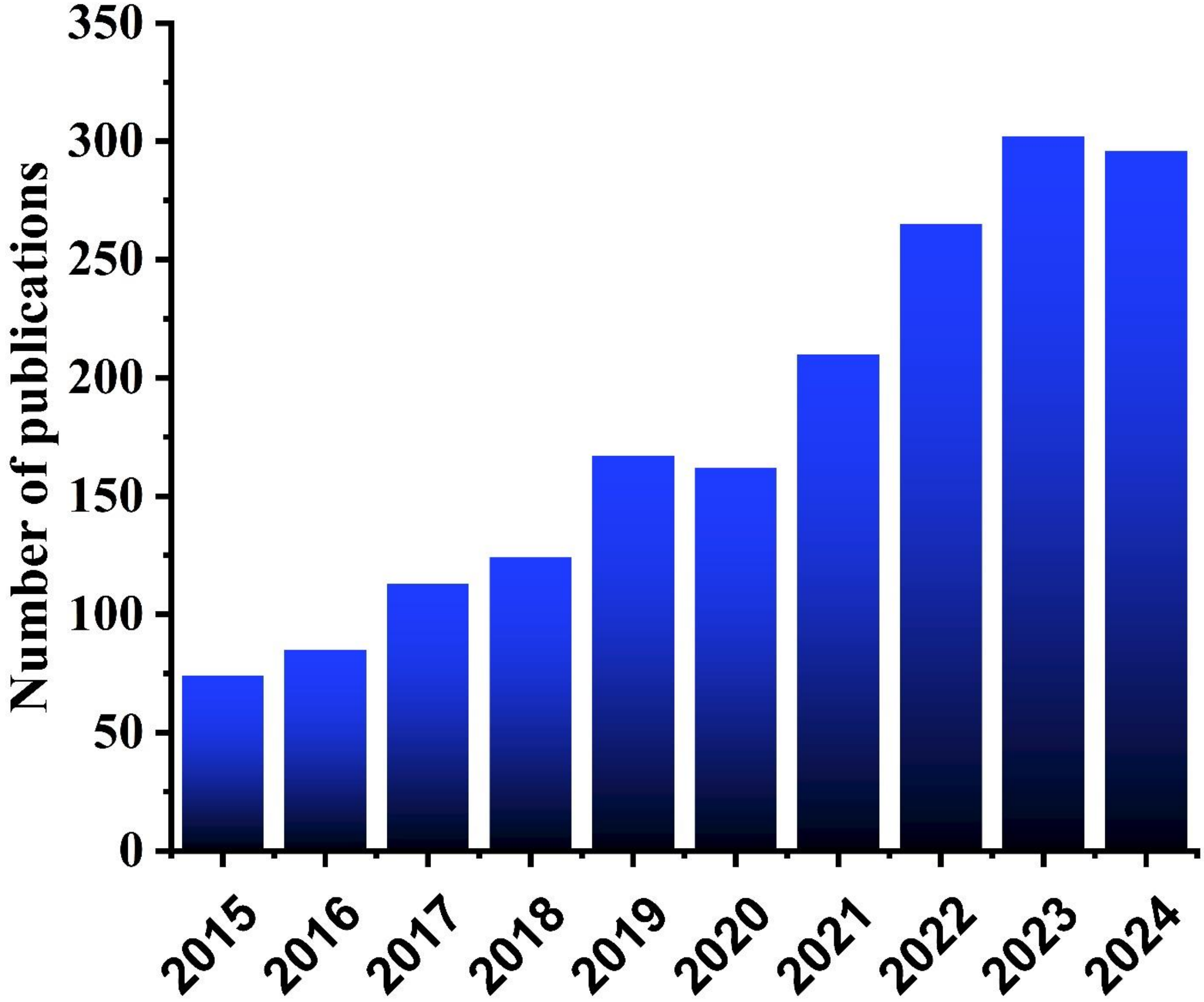

Fig. S2. The number of Scopus-indexed publications related to the filler-loaded membranes applicable in lithium-ion batteries.
